# Alzheimer’s disease-specific transcriptomic and epigenomic changes in the tryptophan catabolic pathway

**DOI:** 10.1186/s13195-024-01623-4

**Published:** 2024-11-30

**Authors:** Kyonghwan Choe, Muhammad Ali, Roy Lardenoije, Renzo J.M. Riemens, Ehsan Pishva, Horst Bickel, Siegfried Weyerer, Per Hoffmann, Michael Pentzek, Steffi Riedel-Heller, Birgitt Wiese, Martin Scherer, Michael Wagner, Diego Mastroeni, Paul D. Coleman, Alfredo Ramirez, Inez H.G.B. Ramakers, Frans R.J. Verhey, Bart P.F. Rutten, Gunter Kenis, Daniel L.A. van den Hove

**Affiliations:** 1 Department of Psychiatry and Neuropsychology, Faculty of Health, Medicine and Life Sciences (FHML), Mental Health and Neuroscience Research Institute (MHeNs) and European Graduate School of Neuroscience (EURON), Maastricht University, Maastricht, 6211 LK the Netherlands; 2 Computational Biology Group, Centre for System Biomedicine (LCSB), University of Luxembourg, Belvaux, Luxembourg; 3 Biomedical Data science Group, Centre for System Biomedicine (LCSB), University of Luxembourg, Belvaux, Luxembourg; 4 Pattern Recognition and Bioinformatics, Department of Intelligent Systems, Delft University of Technology, Delft, the Netherlands; 5 University of Exeter Medical School, University of Exeter, Exeter, UK; 6 Department of Psychiatry, Klinikum rechts der Isar, Technical University of Munich, Munich, Germany; 7 Medical Faculty, Central Institute of Mental Health, Mannheim/Heidelberg University, Heidelberg, Germany; 8 Institute of Human Genetics, University of Bonn, Bonn, 53127 Germany; 9 Department of Genomics, Life & Brain Center, University of Bonn, 53127 Bonn, Germany; 10 Division of Medical Genetics, Department of Biomedicine, University Hospital, University of Basel, CH-4058 Basel, Switzerland; 11 Medical Faculty, Institute of General Practice, Heinrich Heine University Düsseldorf, Düsseldorf, 40225 Germany; 12 Institute of Social Medicine, Occupational Health and Public Health, Leipzig University, Leipzig, 04103 Germany; 13 Work Group Medical Statistics and IT-Infrastructure, Institute for General Practice, Hannover Medical School, Hannover, Germany; 14 Department of Primary Medical Care, Center for Psychosocial Medicine, University Medical Center Hamburg-Eppendorf, Hamburg, Germany; 15 Department of Neurodegeneration and Gerontopsychiatry, University of Bonn, 53127 Bonn, Germany; 16 German Center for Neurodegenerative Diseases (DZNE), 53127 Bonn, Germany; 17 L.J. Roberts Center for Alzheimer’s Research, Banner Sun Health Research Institute, Sun City, AZ USA; 18 Biodesign Institute, Neurodegenerative Disease Research Center, Arizona State University, Tempe, AZ USA; 19 Division of Neurogenetics and Molecular Psychiatry, Department of Psychiatry and Psychotherapy, Medical Faculty, University of Cologne, Cologne, 50937 Germany; 20 Excellence Cluster on Cellular Stress Responses in Aging-Associated Diseases (CECAD), University of Cologne, Cologne, Germany; 21 Department of Psychiatry, and Glenn Biggs Institute for Alzheimer’s and Neurodegenerative Diseases, San Antonio, TX USA; 22 Department of Psychiatry and Neuropsychology, Alzheimer Center Limburg, Maastricht University, Maastricht, the Netherlands

**Keywords:** Tryptophan (TRP), Alzheimer’s disease (AD), Epigenetics, Brain, Blood, Indoleamine 2,3-dioxygenase (IDO)

## Abstract

**Background:**

Neurodegenerative disorders, including Alzheimer’s disease (AD), have been linked to alterations in tryptophan (TRP) metabolism. However, no studies to date have systematically explored changes in the TRP pathway at both transcriptional and epigenetic levels. This study aimed to investigate transcriptomic, DNA methylomic (5mC) and hydroxymethylomic (5hmC) changes within genes involved in the TRP and nicotinamide adenine dinucleotide (NAD) pathways in AD, using three independent cohorts.

**Methods:**

DNA derived from post-mortem middle temporal gyrus (MTG) tissue from AD patients (*n* = 45) and age-matched controls (*n* = 35) was analyzed, along with DNA derived from blood samples from two independent cohorts: the German Study on Ageing, Cognition, and Dementia in Primary Care Patients (AgeCoDe) cohort (*n* = 96) and the Dutch BioBank Alzheimer Center Limburg (BBACL) cohort (*n* = 262). Molecular profiling, including assessing mRNA expression and DNA (hydroxy)methylation levels, was conducted using HumanHT-12 v4 Expression BeadChip and HM 450 K BeadChip arrays, respectively. Functional interactions between genes and identification of common phenotype-specific positive and negative elementary circuits were conducted using computational modeling, i.e. gene regulatory network (GRN) and network perturbational analysis. DNA methylation of *IDO2* (cg11251498) was analyzed using pyrosequencing.

**Results:**

Twelve TRP- and twenty NAD-associated genes were found to be differentially expressed in the MTG of AD patients. Gene sets associated in the kynurenine pathway, the most common TRP pathway, and NAD pathway, showed enrichment at the mRNA expression level. Downstream analyses integrating data on gene expression, DNA (hydroxy)methylation, and AD pathology, as well as GRN and network perturbation analyses, identified *IDO2*, an immune regulatory gene, as a key candidate in AD. Notably, one CpG site in *IDO2* (cg11251498) exhibited significant methylation differences between AD converters and non-converters in the AgeCoDe cohort.

**Conclusion:**

These findings reveal substantial transcriptional and epigenetic alterations in TRP- and NAD-pathway-associated genes in AD, highlighting *IDO2* as a key candidate gene for further investigation. These genes and their encoded proteins hold potential as novel biomarkers and therapeutic targets for AD.

**Supplementary Information:**

The online version contains supplementary material available at 10.1186/s13195-024-01623-4.

## Introduction

 Alzheimer’s disease (AD) is a heterogeneous neurodegenerative disorder characterized by gradual cognitive decline and often displaying coexisting metabolic dysfunction. AD is the most common type of dementia, contributing to 50–75% of cases [1]. The biggest risk factor for dementia is aging [2]. Currently, there is no treatment for AD and the exact cause and pathophysiology remain unclear. While the amyloid beta (Aβ) and tau hypotheses have been investigated for decades, recent studies have shown that other mechanisms could be involved in the development and course of AD as well. For example, recent work has shown that the tryptophan (TRP) catabolic pathway may contribute to neurodegenerative disorders such as AD [3]. TRP is an essential amino acid, supplied only through diet or supplements. TRP competes with other large neutral amino acids for the same large amino acid transporter (LAT) to cross the blood-brain barrier (BBB) [4]. Once TRP enters the brain, it acts as a precursor for many pathways centered around molecules such as kynurenine (KYN), serotonin, tryptamine, and protein synthesis [5].

Current studies on the TRP catabolic pathway largely focus on the kynurenine pathway (KP) as it is the dominant pathway, accounting for > 90% of tryptophan metabolism. Tryptophan 2,3-dioxygenase 2 (TDO2), indoleamine 2,3-dioxygenase 1 (IDO1), and IDO2 are the first and rate-limiting enzymes that metabolize TRP and initiate the KP [6]. Although TDO2 is mainly expressed in the liver, it is also present in the brain. Astrocytes, microglia, microvascular endothelial cells, and macrophages are the main cell types expressing IDO [5]. Further downstream KP metabolites, also known as kynurenines, have shown to exert both neurotoxic (e.g. quinolinic acid [QA]) and neuroprotective (e.g. kynurenic acid [KA]) effects in the brain [4, 5]. With respect to the pathophysiology of AD, KA is considered neuroprotective because it is an antagonist for ionotropic glutamate receptors as well as the α7 nicotinic acetylcholine (α7nACh) receptor [3]. On the contrary, QA is considered neurotoxic because it is an agonist of N-methyl-D-aspartate (NMDA) receptor with the potential to induce excitotoxicity [3]. In cross-sectional studies, plasma KA concentrations were shown to be decreased [7, 8], while QA concentrations were increased in AD patients compared to controls [7]. Additionally, in post-mortem brain studies, the highest QA expression was shown in the perimeter of senile plaques in the hippocampus [9, 10]. Additionally, QA was co-localized with hyperphosphorylated tau within cortical neurons in AD brain and QA treatment increased tau phosphorylation in human primary neurons [11]. The KP also initiates the *de novo* synthesis of nicotinamide adenine dinucleotide (NAD) through QA. NAD is a coenzyme central to metabolism, in particular in view of adenosine triphosphate (ATP) production. Studies have shown a decrease in NAD levels during normal aging, but also during neurodegeneration [12].

Studies systematically investigating the TRP catabolic pathway are lacking. Most studies have used concentrations of multiple metabolites or their ratio to deduce KP activity. While these studies assessing TRP- and NAD-pathway-associated metabolite levels provide valuable information, investigating changes in DNA methylation and mRNA expression of genes linked to these pathways may be of great added value given their crucial role in regulating the pathogenesis of AD and its molecular regulation. Therefore, the aim of the current study was to examine to which extent TRP- and NAD-associated genes were affected in AD at the transcriptional level as well as at the DNA methylation (5-methylcytosine [5mC]), DNA hydroxymethylation (5-hydroxymethylcytosine [5hmC]), and unmodified cytosine [5uC]) levels. For this purpose, we first investigated TRP- and NAD-pathway-associated genes through transcriptomic- and DNA (hydroxy)methylation- profiling, making use of human post-mortem middle temporal gyrus (MTG) tissue from AD patients and controls. This brain region was chosen because the MTG is recognized as an area affected early in AD pathology [13], and prior studies have reported differences in global DNA methylation and hydroxymethylation levels in this region in AD [14]. Subsequent gene regulatory network (GRN) and network perturbation analysis aided us in selecting *IDO2* as a candidate gene for further validation, making use of DNA derived from blood in two independent longitudinal aging cohorts.

## Materials and methods

### Post-mortem MTG brain tissue

Detailed information about the MTG datasets can be found elsewhere [15]. Briefly, MTG DNA samples were obtained from AD patients (*n* = 45) and neurologically normal control (*n* = 35) from Brain and Body Donation Program (BBDP) donors and stored at the Brain and Tissue Bank of the Banner Sun Health Research Institute (BSHRI; Sun City, Arizona, USA) (Table 1). Detailed information about the BBDP has been reported elsewhere [16, 17]. The organization of the BBDP allows for fast tissue recovery after death and samples in this study had an average post-mortem interval (PMI) of 2.8 h. Additionally, Braak staging was carried out for assessing the degree of neurofibrillary pathology. Histopathological scoring (i.e. amyloid plaque and neurofibrillary tangle density) was assessed and categorized across key regions of the brain, including the frontal, temporal, parietal, and occipital lobes, as well as the hippocampus and entorhinal cortex. This evaluation was based on the combined observations from 80 μm brain sections, stained using thioflavin S, Campbell-Switzer, and Gallyas techniques [16]. A consensus diagnosis of AD or non-demented control was reached by following National Institutes of Health (NIH) AD Center criteria [17]. Exclusion criteria were comorbidity with any other type of dementia, cerebrovascular disorders, mild cognitive impairment (MCI), and presence of non-microscopic infarcts. Informed consent was obtained from all human participants. This includes donors of the BSHRI-BBDP, who signed an Institutional Review Board-approved informed consent form, including specific consent to the use of donated tissue for future research [16, 17].

**Table 1:** MTG patient demographics

	AD patients	Controls
N	45	35
Gender (male/female)	22/23	17/18
Age of death	85.09 ± 6.24	84.46 ± 5.50
PMI	2.77 ± 0.69	2.87 ± 1.03
Plaque total	12.97 ± 2.25	4.65 ± 4.30
Tangle total	11.02 ± 4.16	3.96 ± 2.10
Braak Stage (range (median))	II – VI (V)	I – IV (III)

Overview of patient characteristics in post-mortem middle temporal gyrus (MTG) brains used in this study. Data are presented in n or mean ± SD. Patients were age- and gender- matched and diagnosed with either Alzheimer’s disease (AD) or non-demented controls

### The AgeCoDe cohort

Detailed information about the Ageing, Cognition and Dementia in Primary Care Patients (AgeCoDe) datasets can be found elsewhere [15]. AgeCoDe, a prospective longitudinal study, aims to improve early detection of MCI and dementia in primary care and included 3327 non-demented individuals at baseline [18]. For this study, the dataset published by Lardenoije et al. (2019) was used [15]. Briefly, a subsample of 96 individuals (age > 70 years) with whole blood DNA methylation data available, were selected. Of these, 41 individuals converted to AD during the study, while 42 control subjects did not show cognitive impairment at baseline nor follow-up 4.5 years later. The remaining 13 individuals did convert, but only after 4.5 years (Table 2). The groups were matched for age, gender, and *APOE* genotype. Dementia presence was evaluated in all participants using the Structured Interview for the Diagnosis of Dementia of the Alzheimer Type, Multi-Infarct Dementia, and Dementia of Other Etiologies [19], following DSM-IV criteria. For subjects who were not personally interviewed, dementia diagnosis was determined using the Global Deterioration Scale [20] (≥ 4) and the Blessed Dementia Rating subscales. The etiological diagnosis of AD was made according to the criteria of the National Institute of Neurological and Communicative Disorders and Stroke and the Alzheimer’s Disease and Related Disorders Association [21] for probable AD, and was only assigned if sufficient information was available. All final diagnoses were reached by consensus between the interviewer and an experienced geriatrician or geriatric psychiatrist. The German AgeCoDe study protocol was approved by the local ethics committees at the University of Bonn (Bonn, Germany), the University of Hamburg (Hamburg, Germany), the University of Düsseldorf (Düsseldorf, Germany), the University of Heidelberg/Mannheim (Mannheim, Germany), the University of Leipzig (Leipzig, Germany), and the Technical University of Munich (Munich, Germany).

**Table 2:** AgeCoDe cohort patient demographics

	Controls	AD Converters
**Baseline (T1)**		
N	42	54
Age at baseline	81.00 ± 3.11	82.31 ± 3.55
Gender (male/female)	10/32	17/37
* IDO2* (cg11251498) (%)	72.53 ± 5.36	75.59 ± 3.71
**Follow-up (T2)**		
N	42	41
Age at baseline	81.00 ± 3.11	82.01 ± 3.51
Gender (male/female)	10/32	13/28
* IDO2* (cg11251498) (%)	73.07 ± 4.91	75.25 ± 4.96

Overview of patient characteristics in the AgeCoDe cohort used in this study. Data are presented in n or mean ± SD. Patients were age- and gender-matched. Blood was collected at baseline and follow-up (average 4.5 years). All patients were controls at baseline and the patients were followed-up over time

### The BBACL cohort

Detailed information about the Biobank Alzheimer Center Limburg (BBACL) datasets can be found elsewhere [22]. BBACL study is an ongoing, prospective clinical cohort of patients referred to the Memory Clinic of the Maastricht University Medical Center + (MUMC+), the Netherlands, for the evaluation of their cognitive complaints. These patients were diagnosed either with subjective cognitive decline (SCD), mild cognitive impairment (MCI), or dementia. Inclusion criteria were a clinical dementia rating scale (CDR; Morris 1993) score from 0 to 1, and a Mini-Mental State Examination (MMSE; Folstein 1975) score ≥ 20, thereby including patients across the clinical spectrum of SCD, MCI and mild dementia. Exclusion criteria at baseline were non-degenerative neurological diseases, i.e. Normal Pressure Hydrocephalus, Huntington’s disease, brain tumor, epilepsy, encephalitis, recent transient ischemic attack (TIA) or cerebrovascular accident (CVA) (< 2 years), or TIA/CVA with concurrent (within three months) cognitive decline; a history of psychiatric disorders, current major depressive disorder (within 12 months) (DSM IV), or alcohol abuse. All patients underwent a physical, cognitive and neuropsychiatric evaluation and biomaterials were collected. SCD and MCI patients were followed-up over time and a proportion developed dementia. For this study, individuals were selected based on the availability of baseline DNA samples. As such, DNA (hydroxy)methylation levels were measured using pyrosequencing of DNA isolated from whole blood samples from 262 individuals: SCD (*n* = 39), MCI (*n* = 168), and dementia (*n* = 55). Amongst the 168 MCI patients, 80 patients developed dementia (MCI-D) while 88 individuals remained MCI (MCI-MCI) within 76 months after baseline (Table 3). The BBACL study protocol was approved by local ethics committees (METC 15-4-100) at the MUMC+ (Maastricht, the Netherlands). All participants gave their written informed consent.

**Table 3:** BBACL cohort patient demographics

	SCD	MCI	Dementia	MCI-D	MCI-MCI
**Demographics variables**					
N	39	168	55	80	88
Age at baseline	59.62 ± 9.50	72.65 ± 7.69	74.44 ± 7.89	74.94 ± 6.24	70.57 ± 8.30
Gender (male/female)	33/6	89/79	24/31	45/35	44/44
Education (low/middle/high)	13/16/10	64/66/38	27/20/8	26/31/23	38/35/15
**Lifestyle variables**					
BMI (kg/m^2^)	25.95 ± 4.00 (*n* = 22)	26.66 ± 4.17 (*n* = 121)	24.67 ± 3.97 (*n* = 42)	26.28 ± 3.67 (*n* = 58)	27.01 ± 4.57 (*n* = 63)
Smoking status (never/< 6 months/ > 6 months/current)	17/0/13/8	74/1/61/22	30/0/17/4	38/0/32/7	36/1/29/15
Alcohol consumption (yes/no)	30/6	116/42	38/12	60/17	56/25
**Cognitive test**					
MMSE	28.31 ± 1.58	26.65 ± 2.30	24.07 ± 2.28	26.03 ± 2.42	27.23 ± 2.04
normMMSE	80.52 ± 14.04	68.41 ± 15.63	53.26 ± 11.03	64.38 ± 15.30	72.07 ± 15.10
**Pyrosequencing (%)**					
* IDO2* (cg11251498)	64.96 ± 4.55	63.31 ± 5.12	63.91 ± 6.06	63.39 ± 5.93	63.23 ± 4.28

Overview of patient characteristics in the BBACL cohort used in this study. Data are presented in n or mean ± SD

*Abbreviations:* *SCD* subjective cognitive decline, *MCI* mild cognitive impairment, *MCI-D* MCI to dementia converters, *MCI-MCI* MCI to MCI non-converters, *MMSE* mini mental state examination, *normMMSE* normalized mini mental state examination

### Identification of tryptophan catabolic pathway-associated genes

The TRP catabolic pathway can be broadly divided into two main pathways, i.e., the TRP (metabolic) pathway which includes the KYN, serotonin, tryptamine, and protein synthesis sub-pathways, and the NAD pathway which includes the *de novo* biosynthesis, Salvage, and Preiss-Handler sub-pathways. Our lists of genes of the TRP- and NAD- pathways were generated by combining three databases: the Kyoto Encyclopedia of Genes and Genomes (KEGG), WikiPathways, and Reactome. In the KEGG database, the *Homo sapiens* “tryptophan metabolism pathway” (pathway: hsa00380) [23] and the “nicotinate and nicotinamide metabolism pathway” (pathway: hsa00760) [24] were selected. In WikiPathways, the “Tryptophan metabolism pathway (*Homo sapiens*)” from Lynn M. Ferrante et al. [25], and the “NAD biosynthetic pathways (*Homo sapiens*)” from Kristina Hanspers et al. [26], were selected. Finally, in Reactome, “Tryptophan catabolic” (Identifier: R-HSA-71240) [27], “Serotonin and melatonin biosynthesis” (Identifier: R-HSA-209931) [28], and “Nicotinate metabolism” (Identifier: R-HSA-196807) [29] were selected. This systematic search was validated and complemented by screening the available scientific literature on this matter (Table S1 and S2).

### Transcriptomic- and DNA (hydroxy)methylomic- profiling

The brain tissue samples used for RNA extraction were identical to those used in the DNA methylation study. Both transcriptomic and DNA (hydroxy)methylomic data were obtained from a previous study by our group, as published by Lardenoije et al. (2019) [15]. The gene expression microarray data used in the present study was generated using Illumina HumanHT-12 v4 BeadChip arrays as described in more detail previously [30]. For differential DNA methylation analysis in the BSHRI-BBDP samples, bisulfite (BS) and oxidative BS (oxBS) conversion of genomic DNA (gDNA) derived from MTG tissue was performed using the TrueMethyl 24 Kit version 2.0 (Cambridge Epigenetix, Cambridge, UK). A total of 8µL from each BS/oxBS-treated DNA sample was amplified and hybridized on HM 450 K arrays (Illumina, Inc., San Diego, CA, USA) for quantifying methylation status of different human ‘5-Cytosine-phosphate-Guanine-3’ (CpG) sites. All procedures were performed according to the manufacturer’s protocol. For the AgeCoDe samples, gDNA was isolated from whole blood and concentration was measured using the NanoDrop ND1000 spectrophotometer (Thermo Fisher Scientific, Waltham, MA, USA). Then, 200ng BS-treated DNA was analyzed using HM 450 K arrays according to the manufacturer’s protocol. Imaging of the arrays was done using the Illumina iScan.

#### Bisulfite conversion

DNA samples of 310 BBACL individuals were isolated from the buffy coat using the QIAsymphony DSP DNA Midi kit (QIAGEN, Hilden, Germany) following the manufacturer’s instructions. Isolated DNA was aliquoted and stored at −80 °C for later use. The DNA concentration was measured using the Qubit dsDNA BR Assay Kit (Q32853, Thermo Fisher Scientific) and Qubit 2.0 Fluorometer (Thermo Fisher Scientific) analytical instrument, following the manufacturer’s instructions. In the end, 262 DNA samples met the minimal requirement of 200ng to be used as input for BS treatment. For this purpose, DNA was BS-treated using the EZ-96 DNA Methylation-Gold kit (D5008, ZYMO RESEARCH), following the manufacturer’s instructions with one minor adjustment in which the BS-converted samples were eluted in 20µL of elution buffer. One µL of the resulting sample was used for polymerase chain reaction (PCR) amplification followed by BS pyrosequencing. All BS conversion assays included at least two negative controls.

#### PCR

*IDO2* (cg11251498) PCR and pyrosequencing primers (reverse direction) were designed using the PyroMark Assay Design version 2.0.1.15 (QIAGEN) (Table S3) using Ensembl Genome Browser GRCh37 assemble database. All PCR reactions used FastStart™ Taq DNA Polymerase, dNTPack (Roche Diagnostics GmbH, Mannheim, Germany) following manufacturer’s instructions. Briefly, each PCR reaction contained 2.5µL of PCR buffer (10x) with 20mM MgCl_2_, 0.5µL of dNTPs, 1µL of forward and reverse primers (5µM), 0.2µL of FastStart Taq DNA polymerase (5U/µL), and 1µL of BS treated gDNA in a total volume of 25µL. All PCR reactions had two positive and negative (BS negative and water each) controls. In addition, the PCR reaction was performed as follows: denaturation at 95 °C for 5 min; 58 cycles of 95 °C for 30 s, 57 °C for 30 s, 72 °C for 30 s; followed by final extension for 1 min at 72 °C. Each PCR product (150 bp) was then size-fractionated on a 2% agarose gel in Tris-Acetate-EDTA (TAE) buffer.

#### Pyrosequencing

Pyrosequencing was performed and quantified using the PyroMark Q48 Autoprep system and Pyro Q48 Autroprep 2.4.2 software (QIAGEN) following the manufacturer’s instructions. The sensitivity of the assay was assessed using methylated and unmethylated DNA standards from the EpiTect PCR Control DNA set (QIAGEN). All pyrosequencing runs included multiple negative controls and only samples that passed the quality control were included.

### Computational modeling

A detailed description of the protocol for the gene regulatory network (GRN) and network perturbation analysis has been published elsewhere [31]. Briefly, functional interactions between genes in the TRP- and NAD-associated pathways were identified through MetaCore (Clarivate Analytics, London, UK). MetaCore is a collection of manually curated and experimentally validated direct gene-gene interactions. The analysis was restricted to “Functional interactions”, “Binding interactions”, and “Low trust interactions” to build an interaction network map pinpointing functional interactions among the selected genes.

A network perturbation analysis is a tool to identify common and phenotype-specific positive and negative elementary circuits. Studies have reported the significant role of these circuits in both maintaining network stability and the existence of these circuits is considered to be a necessary condition for having stable steady-state [32, 33]. Network perturbation analysis was performed through Java implementations as proposed by Zickenrott et al. [34], in which a network simulation analysis combined single genes and sets of up to four genes, in order to identify and rank genes and gene sets based on their ability to regulate the expression of downstream genes.

### Statistical analysis

A detailed description of the statistical analysis performed for the BSHRI-BBDP and AgeCoDe data can be found elsewhere [15]. All computational and statistical analyses for these two cohorts were performed using R (version 3.3.2) and RStudio (version 1.0.136) [35]. Briefly, raw expression data were log-transformed and quantile-quantile normalized. For computing the cell composition, the “Neun_pos” (neuronal positive) cell percentage was calculated from the methylation data. The same regression model used for assessing methylation was applied to the expression data where the effects of age, gender and cell type composition were regressed out using limma [31]. Additionally, a false discovery rate (FDR) correction for multiple testing was applied for both transcriptomic- and DNA (hydroxy)methylomic- profiling in which a *q*-value less than 0.05 was considered statistically significant.

All remaining statistical analyses for BBACL and AgeCoDe (only for pyrosequencing and age association analyses) were performed using SPSS Statistics version 27. For BBACL, crude mini mental state exam (MMSE) scores (range 0–30) were normalized (normMMSE; range 0–100) using the NormPsy (version 1.0.7) function. Normalization preserves the ranking of the test scores but adjusts the gaps between consecutive values to correct the curvilinearity of the MMSE. Additionally, different models were performed to adjust for the effect of covariates using general linear model univariate analysis, in which *model 1* adjusted for age, gender, education, and *model 2* (only for BBACL) additionally adjusted for lifestyle (smoking status, drinking status, and body mass index (BMI)). *Model 1* was considered the main model. All association studies reported the beta value (β), standard error (S.E.), *p*-value, and 95% confidential interval (95% CI). *P*-values less than 0.05 were considered statistically significant.

## Results

### Altered gene expression profiles in the brain of patients with AD

**Fig. 1:**
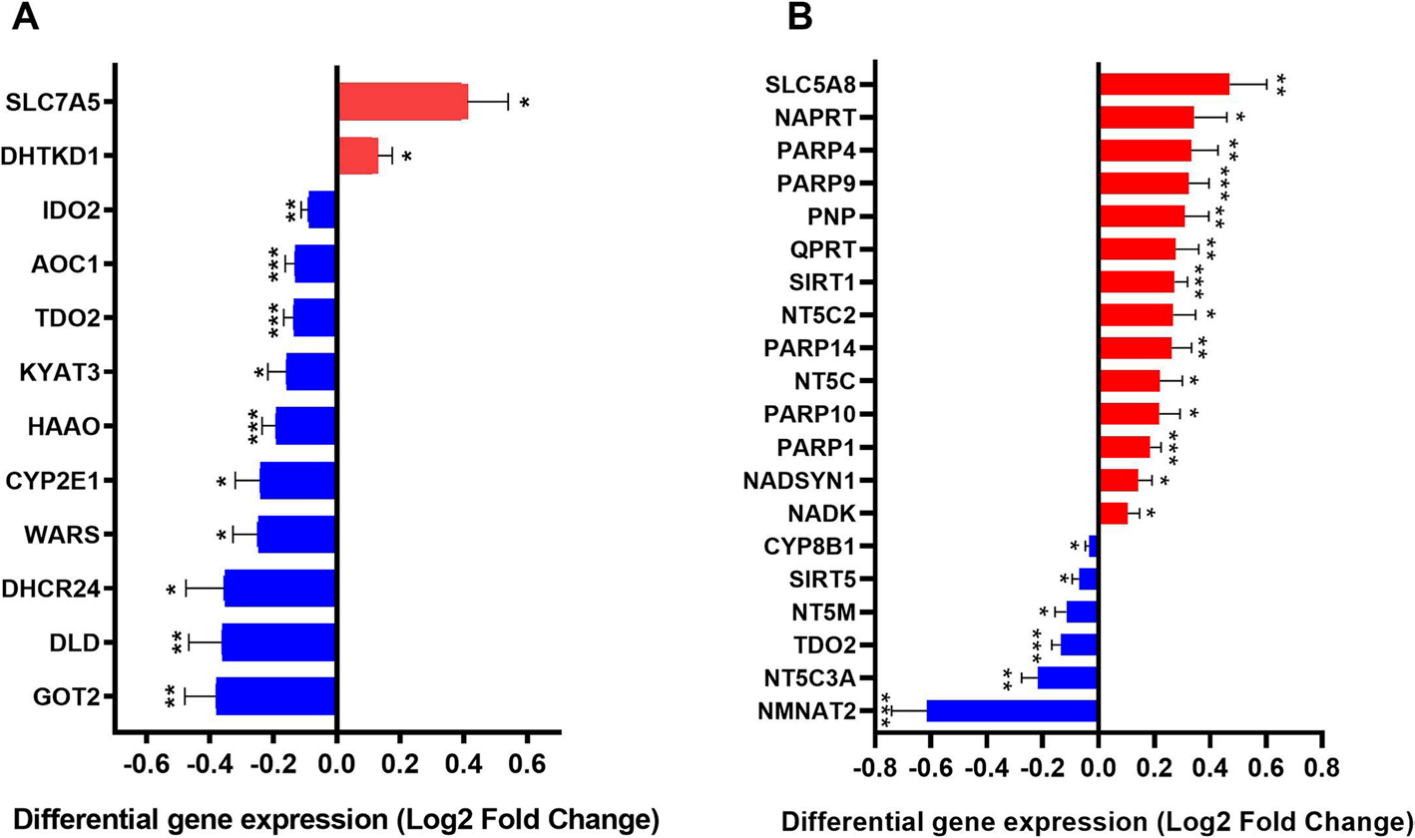
TRP and NAD pathway associated genes show significant differential gene expression. Shown are all the genes in the TRP and NAD pathway with up (red) or down (blue) regulation in the middle temporal gyrus of AD patients compared to age-matched controls. Differential expression is presented as log2 fold change. Differential regulation was assessed using false discovery rate (FDR) test and q < 0.05 was considered significant. * *q* < 0.05, ** *q* < 0.01 and *** *q* < 0.001. Error bars represent standard error of mean (SEM)

Based on the systematic research of three databases, it resulted in a gene set consisting of 60 TRP- and 57 NAD-associated genes (Tables S1 and S2). Out of the 60 identified TRP pathway-associated genes, 56 genes were included in the microarray analysis. After FDR correction, 12 of these genes (2 up-regulated [SLC7A5, DHTKD1] and 10 down-regulated [TDO2, HAAO, AOC1, GOT2, IDO2, DLD, SLC7A5, WARS, CYP2E1, DHCR24, KYAT3]) showed significant differential mRNA expression in patients with AD versus controls (Fig. 1A; Table S4). In addition, microarray data were available for 52 of the 57 NAD pathway-associated genes. After FDR correction, mRNA levels of 20 genes showed statistically significant differences with 6 genes showing lower expression (CYP8B1, SIRT5, NT5M, TDO2, NT5C3A, NMNAT2) and 14 genes showing higher expression (SLC5A8, NAPRT, PARP4, PARP9, PNP, SIRT1, QPRT, PARP14, NT5C2, PARP10, NT5C, PARP1, NADSYN1, NADK) (Fig. 1B; Table S5).

### Transcriptomic enrichment analysis

To examine whether expression changes in TRP- and NAD-related genes are overrepresented in AD, an enrichment analysis was conducted based on the transcriptomic profiling within the MTG. It identified 31,726 genes of which 11,459 genes were identified to be nominally (limma *p*-value < 0.05) differentially expressed between AD patients and age-matched control individuals [15]. Once adjusting for multiple testing (limma FDR *q*-value < 0.05), 7,776 genes were significantly differentially expressed at the mRNA level, resulting in an overall enrichment score of 24.5% (7,776/31,726 genes) as a baseline. The overall TRP pathway only displayed a 21.4% enrichment score (12/56 genes), indicating this gene set overall did not display a significant enrichment in view of differentially expressed genes (Table S4). However, the enrichment score for the KP, which accounts for > 90% of all TRP metabolism, showed a 41.7% enrichment score (5/12 genes) indicating a significant enrichment in differentially expressed genes within the KP-associated gene set (Table S4). Lastly, the NAD pathway showed a 38.5% enrichment score (20/52 genes) indicating a significant enrichment in differentially expressed genes within the NAD-associated gene set (Table S5). NAD pathway is divided into two sub-pathways, Salvage pathway and Preiss-Handler pathway, and both pathways were enriched (Salvage: 34.14% [14/41 genes]; Preiss-Handler: 50% [8/16 genes]).

### Correlation between gene expression and AD pathologies

Correlation analysis was conducted to assess the association between mRNA expression and AD pathologies (total Aβ plaque and tau tangle). Based on the KP-associated genes with multiple test correction, *TDO2*, *GOT2*, and *IDO2* showed significant negative correlation with both plaque and tangle, while *KYAT3* only showed negative correlation with total plaque (Table S6). In a similar manner, the NAD-associated genes, *SIRT1*, *PARP9*, *PARP14*, *QPRT*, *PARP10*, *NAPRT*, *NADSYN1*, and *NADK* showed positive correlation in both plaque and tangle, while *PNP*, *NT5C2*, *PARP1*, and *PARP4* was positively correlated in plaque only. Additionally, *NMNAT2* and *NT5C3A* showed negative correlation in both plaque and tangle (Table S6). Based on these results, several significantly differentiated expressed genes in both KP- and NAD-associated pathways were significantly correlated in AD pathologies even after multiple test corrections.

### Alterations of DNA (hydroxy)methylation in the brain of patients with AD

**Fig. 2:**
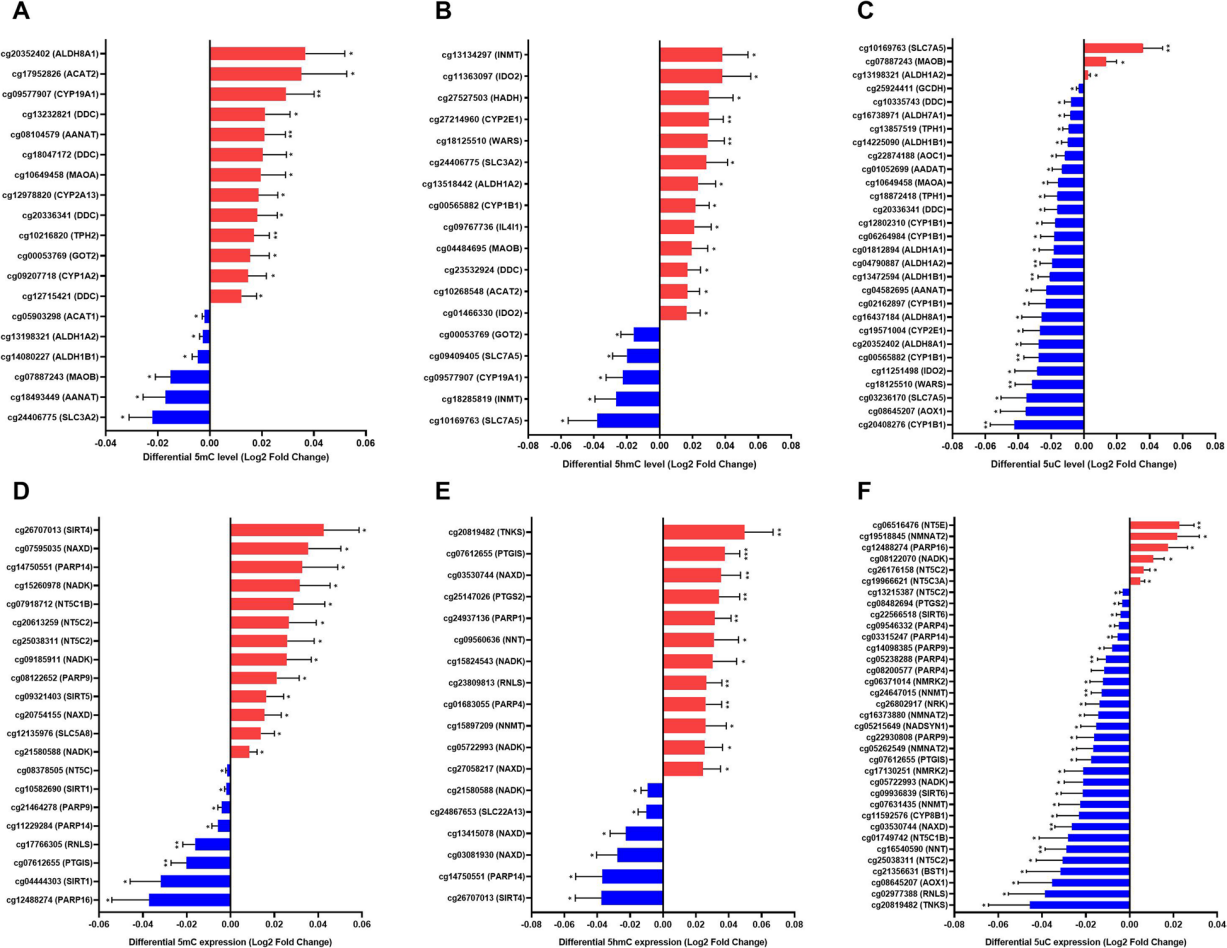
TRP and NAD pathway associated genes show significant differential DNA (hydroxy)methylation levels. Shown are significant CpG sites with its corresponding gene name in the tryptophan metabolic pathway (**A-C**) and NAD pathway (**D-F**) with up (red) or down (blue) regulation in the middle temporal gyrus of AD patients compared to age-matched controls. **A**,** D** Differential methylation (5mC) expression. **(B**,** E)** Differential hydroxymethylation (5hmC) expression. **C**,** F** Differential unmodified (5uC) expression. Differential expression is presented as log2 fold change. Differential regulation was assessed using limma differential DNA (hydroxy)methylation level analysis and *p* < 0.05 was considered significant. * *p* < 0.05 and ** *p* < 0.01. Error bars represent standard error of mean (SEM)

**Fig. 3:**
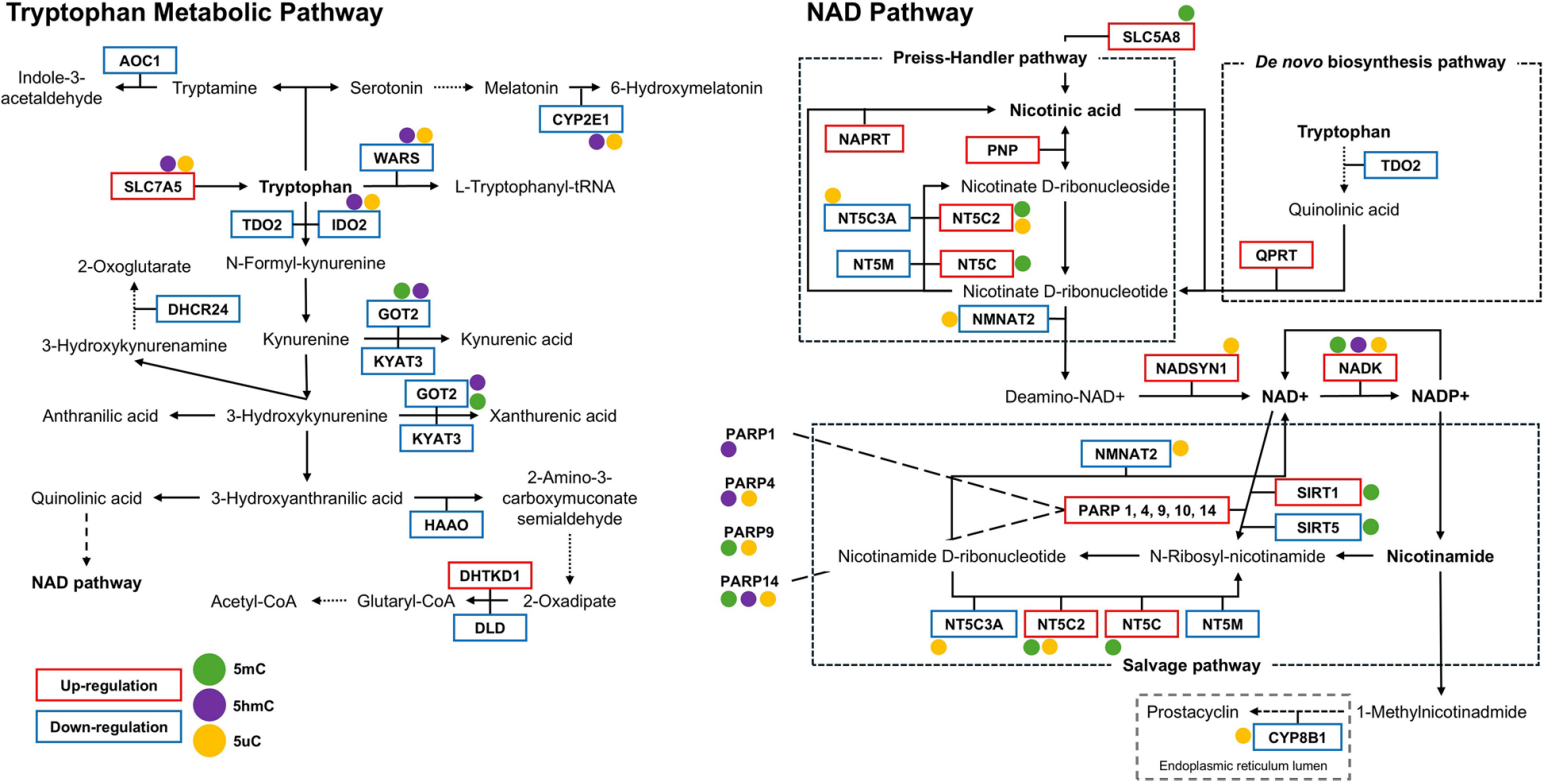
Overview of the differentially expressed genes and DNA (hydroxy)methylation levels within the TRP metabolic pathway and NAD pathway. Within the TRP metabolic pathway, a total of 12 genes exhibited significant differential gene expression, while 20 genes were differentially expressed within the NAD pathway. Genes exhibiting up-regulation in expression are highlighted with red box, whereas those with down-regulation are indicated by blue box. Genes with altered DNA (hydroxy)methylation levels are denoted by circular markers, with distinct colors representing specific modifications: 5mC (green), 5hmC (purple), and 5uC (orange)

Next, we investigated whether the AD-specific TRP- and NAD-associated mRNA profiles were associated with DNA methylation differences at the level of 5mC, 5hmC, or 5uC. Within the TRP pathway, differences in 5mC, 5hmC, and 5uC levels showed nominal significance (*p*-value < 0.05) for 19/847 probes, 18/510 probes, and 29/847 probes, respectively (Fig. 2A-C; Tables S7-S9). Within the NAD pathway, 5mC, 5hmC, and 5uC showed nominally significant differences for 20/1009 probes, 18/632 probes, and 35/1009 probes, respectively (Fig. 2D-F; Tables S10-S12). These differentially (hydroxy)methylated probes were associated with 18 significantly differentially expressed genes. Of these, five genes exhibited significant differences in various forms of methylation (i.e. 5mC, 5hmC, and/or 5uC) at the same CpG site. An overview of the differently expressed genes and DNA (hydroxy)methylation levels within the TRP metabolic pathway and NAD pathway are shown in Fig. 3. It is worth noting that, in the DNA (hydroxy)methylomic profile analyses, the TRP-associated genes *ASMT* and *KYAT1* were not included in this analysis, as data on DNA (hydroxy)methylomic- profiles were not available for these genes. In addition, *ALDH9A1* and *SLC36A4* were excluded from the 5hmC analysis due to missing data. Concerning the NAD pathway, the *NADK2*,* NMNAT1*, and *NMRK* genes were not included in the analysis, as data were not available for these genes. In addition, *PARP2* was excluded from the 5hmC analysis due to missing data.

### Correlation between gene expression and DNA (hydroxy)methylated probes

**Fig. 4:**
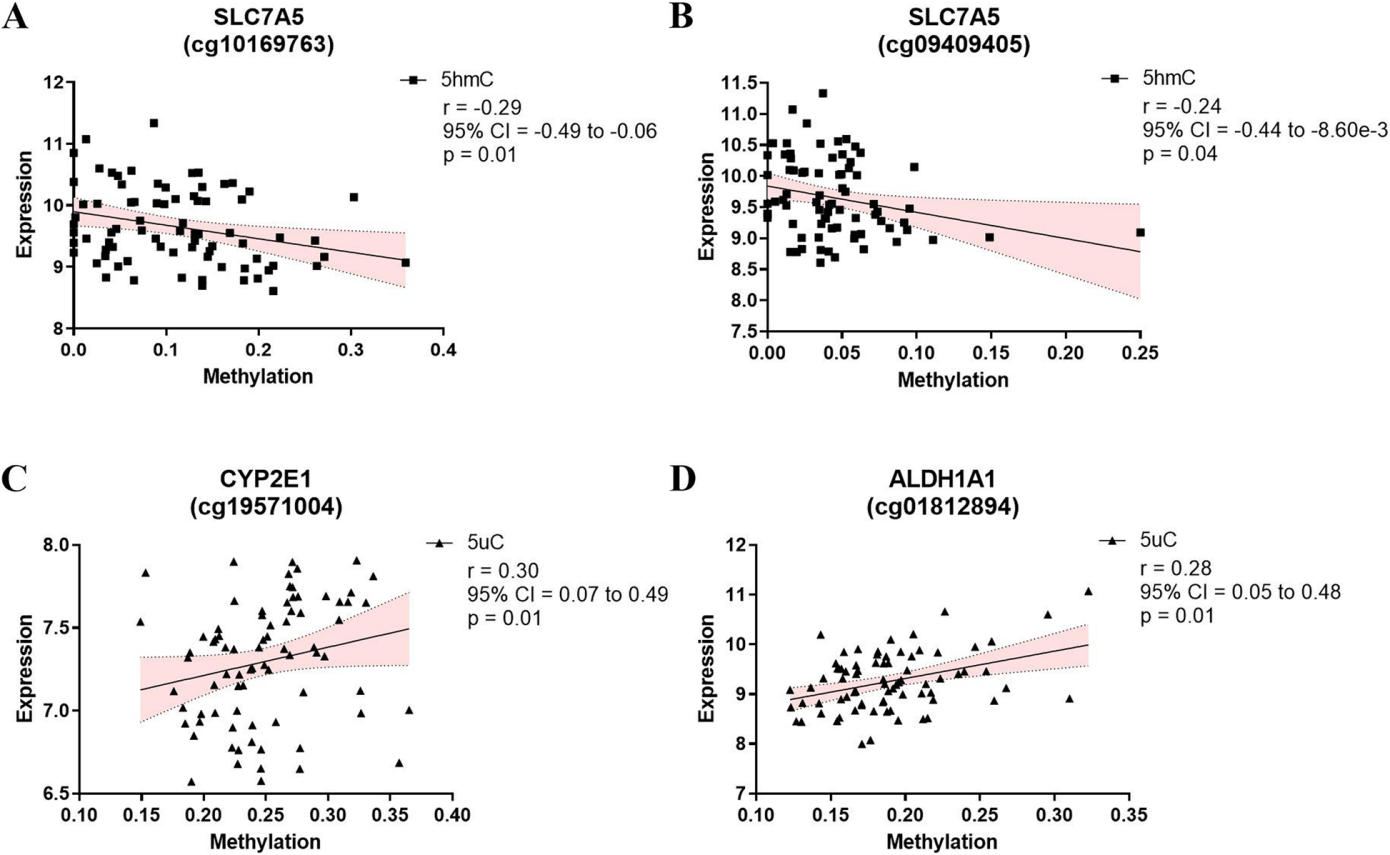
Spearman’s correlation analysis of TRP pathway. Spearman’s correlation analysis between methylation (■5hmC and ▲5uC) and mRNA expression levels. Spearman’s correlation analysis are presented with Spearman’s *R*-value, 95% confidence interval (CI), and *p*-value

**Fig. 5:**
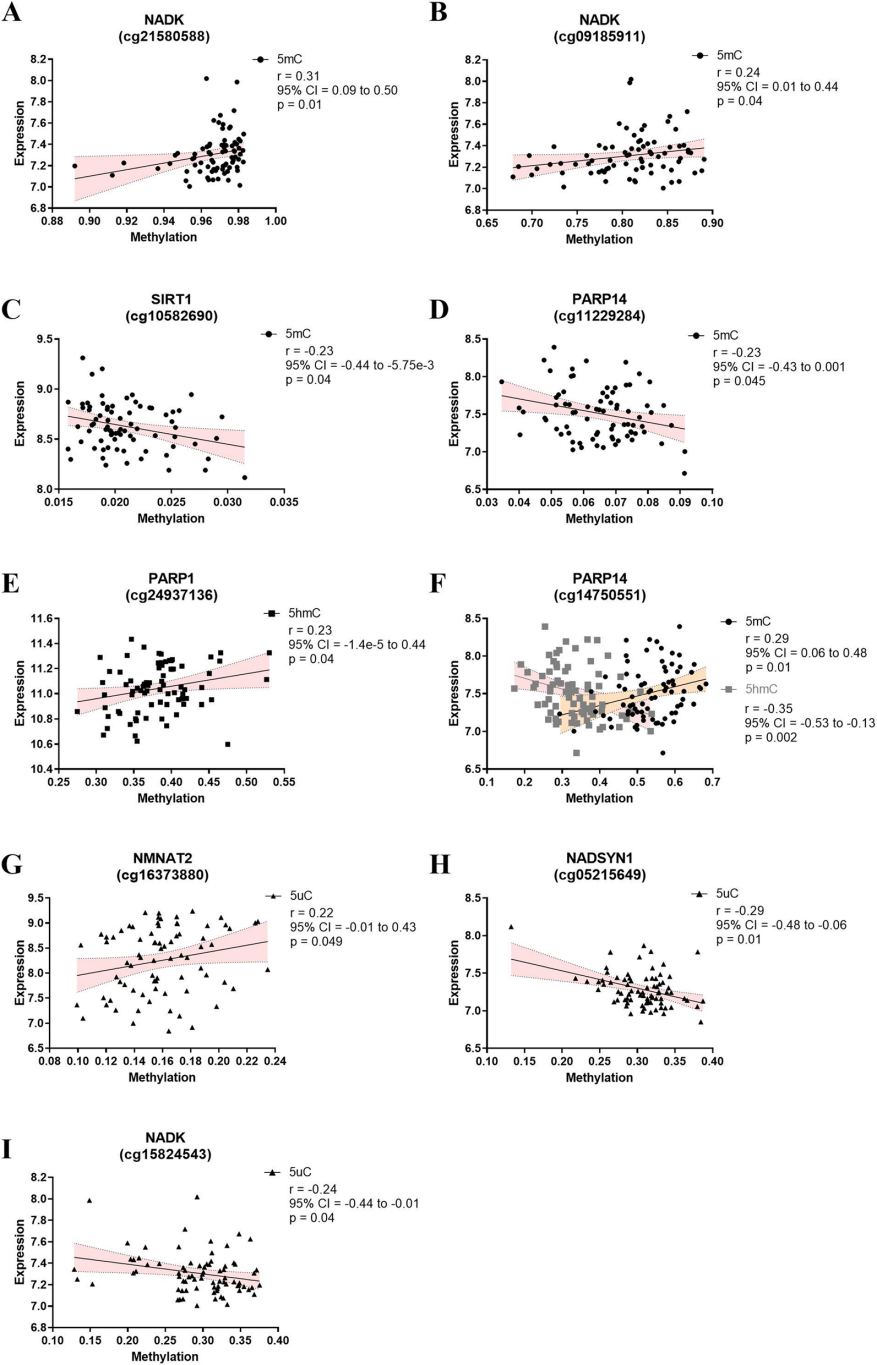
Spearman’s correlation analysis of NAD pathway. Spearman’s correlation analysis between methylation (●5mC, ■5hmC, and ▲5uC) and mRNA expression levels. Spearman’s correlation analysis are presented with spearman *R*-value, 95% confidence interval and, *p*-value

The differentially (hydroxy)methylated probes were linked to several significantly differentially expressed genes. Seven of these genes showed significant differences in different types of methylation modifications for the same CpG site. Moreover, two differentially hydroxymethylated CpG sites within the *SLC7A5* gene, i.e., cg10169763 and cg09409405, displayed a negative correlation between 5hmC levels and mRNA expression (Fig. 4A, B). For 5uC, both cg19571004 (*CYP2E1*) and cg01812894 (*ALDH1A1*) showed a positive correlation with mRNA expression (Fig. 4C, D). In relations to the NAD pathway, nine CpG sites displayed significant correlations between DNA (hydroxy)methylation and mRNA expression (Fig. 5). Concerning 5mC, three CpG sites (cg21580588 [*NADK*]; cg09185911 [*NADK*]; cg14750551 [*PARP14)*]) showed a positive correlation (Fig. 5A, B and F), while two other CpG sites (cg10582690 [*SIRT1*] and cg11229284 [*PARP14*]) showed a negative correlation (Fig. 5C, D). At the 5hmC level, again, cg14750551 (*PARP14*) showed a significant negative correlation (Fig. 5F), while cg24937136 (*PARP1*) was positively correlated (Fig. 5E) to its mRNA expression profile. Finally, for 5uC, cg16373880 (*NMNAT2*) showed a positive correlation (Fig. 5G), whereas two CpG sites (cg05215649 [*NADSYN1*] and cg15824543 [*NADK*]) showed a negative correlation (Fig. 5H, I) with the corresponding mRNA levels.

### Gene regulatory network and network perturbation analysis

**Fig. 6:**
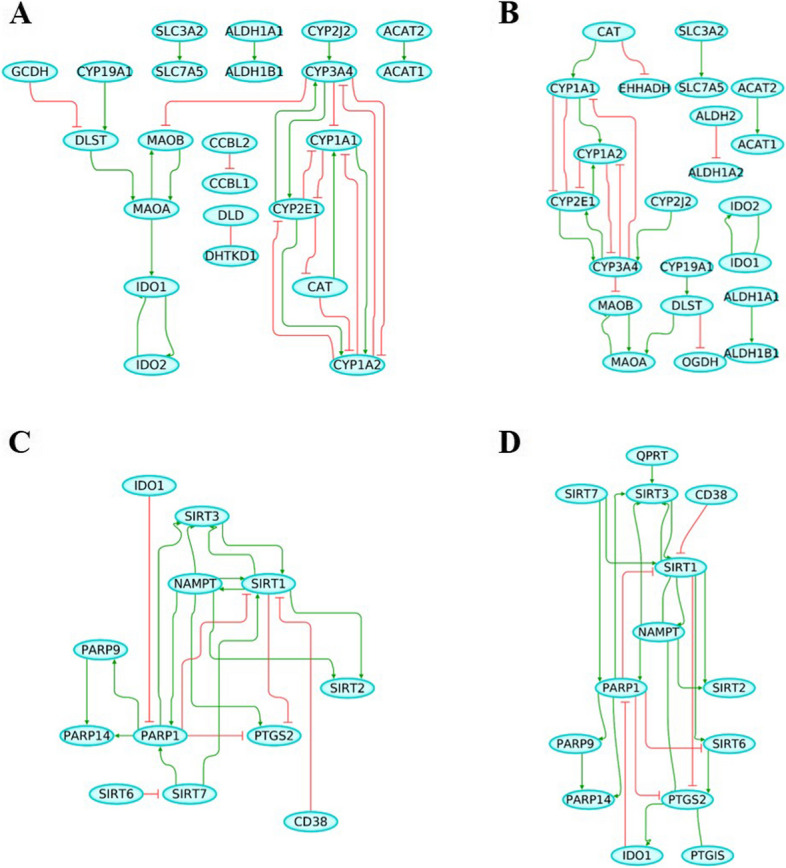
Gene regulatory network (GRN) of TRP and NAD pathway.** A** TRP GRN representing the control phenotype and containing 22 nodes and 30 interactions, (**B**) TRP GRN representing Alzheimer’s disease (AD) phenotype and containing 22 nodes and 25 interactions, (**C**) NAD GRN representing the control phenotype and containing 13 nodes and 21 interactions, (**D**) NAD GRN representing Alzheimer’s disease (AD) phenotype and containing 14 nodes and 24 interactions. Green line indicates gene activation while red line indicates gene inhibition

A GRN was generated through MetaCore (Clarivate Analytics), which builds gene networks by implementing direct functional interactions between genes acquired from experiments-based literature reports. In combination with identifying a GRN, we identified network perturbation candidates that have the potential to induce a positive phenotypic transition, i.e., as in moving from a diseased to a healthy GRN (Table 4). Concerning the TRP pathway, the reconstructed control network comprised 22 nodes and 30 interactions (Fig. 6A), while the AD network comprised of 22 nodes and 25 interactions (Fig. 6B). In the associated perturbation analysis, 9 genes were identified whose alteration holds the potential to revert the gene expression program from a diseased towards a healthy state. Two of these genes (*IDO2* and *CYP2E1*) showed significant changes in differential mRNA expression after FDR correction when comparing AD cases with control subjects. The highest perturbation scores were obtained for a 3-gene perturbation combination, involving *CAT*,* IDO2*, and *CYP2E1* (Table 4).

**Table 4:** Perturbation scores of TRP- and NAD- pathway associated genes

TRP Pathway Genes		NAD Pathway Genes	
Perturbation Score	Combination	Perturbation Score	Combination
10	[CAT, IDO2]	8	[SIRT7, SIRT3]
10	[CAT, IDO1]	8	[IDO1, SIRT3]
9	[MAOA, CAT]	7	[SIRT7, SIRT1]
9	[IDO2, CYP3A4]	7	[SIRT7, PTGS2]
9	[CYP3A4, IDO1]	7	[SIRT1, IDO1]
9	[CYP2E1, IDO2]	7	[NAMPT, SIRT7]
9	[CYP2E1, IDO1]	6	[SIRT7, IDO1]
9	[CYP1A2, IDO2]	6	[PARP1, SIRT7]
9	[CYP1A2, IDO1]	6	[PARP1, SIRT3]
9	[CYP1A1, IDO2]	6	[PARP1, SIRT1]
9	[CYP1A1, IDO1]	6	[PARP1]
9	[CAT]	6	[NAMPT, IDO1]
8	[MAOB, CAT]	5	[SIRT3]
8	[MAOA, CYP3A4]	5	[PTGIS, SIRT3]
8	[MAOA, CYP2E1]	5	[PTGIS, PARP1]
8	[MAOA, CYP1A2]	5	[PARP1, NAMPT]
8	[MAOA, CYP1A1]	5	[PARP1, IDO1]
8	[CYP3A4]	4	[SIRT1, SIRT3]
8	[CYP2E1]	4	[SIRT1]
8	[CYP1A2]	4	[PTGS2, SIRT3]
8	[CYP1A1]	4	[PTGIS, SIRT1]
8	[CAT, CYP3A4]	4	[PTGIS, NAMPT]
8	[CAT, CYP2E1]	4	[PARP1, PTGS2]
8	[CAT, CYP1A2]	4	[NAMPT, SIRT3]
8	[CAT, CYP1A1]	4	[NAMPT]
7	[MAOB, CYP3A4]	3	[PTGS2, SIRT1]
7	[MAOB, CYP2E1]	3	[NAMPT, SIRT1]
7	[MAOB, CYP1A2]	3	[NAMPT, PTGS2]
7	[MAOB, CYP1A1]	2	[PTGIS, IDO1]
7	[CYP2E1, CYP3A4]	2	[PTGIS]
7	[CYP1A2, CYP3A4]	2	[IDO1]
7	[CYP1A2, CYP2E1]	1	[PTGS2, IDO1]
7	[CYP1A2, CYP1A1]	1	[PTGS2]
7	[CYP1A1, CYP3A4]	1	[PTGIS, PTGS2]
7	[CYP1A1, CYP2E1]		
6	[MAOA, IDO2]		
6	[MAOA, IDO1]		
5	[MAOB, IDO2]		
5	[MAOB, IDO1]		
5	[MAOA]		
5	[IDO2]		
5	[IDO1]		
4	[MAOB, MAOA]		
4	[MAOB]		
4	[IDO2, IDO1]		

Overview of network perturbation scores for TRP- and NAD-pathway associated genes. The list of the gene’s full name can be found in the Table S1 and S2

Similarly, GRNs for the NAD pathway were constructed. The control NAD network comprised 13 nodes and 21 interactions (Fig. 6C), while the AD network comprised 14 nodes and 24 interactions (Fig. 6D). Furthermore, in the perturbation analysis, 8 genes were identified, of which 2 genes (*SIRT1* and *PARP1*) showed significant differential mRNA expression after FDR correction. The highest perturbation score was obtained for a 2-gene perturbation combination, involving *SIRT1* and *PARP1* (Table 4).

### *IDO2* methylation in AgeCoDe

Based on the results from the molecular profiles, correlations, and computational models, *IDO2*, *SLC7A5*, and *PARP14* were selected as potential candidate genes for further investigation. As such, an independent longitudinal cohort (AgeCoDe), with DNA derived from blood available, was used in an attempt to validate the MTG methylation findings. In the AgeCoDe cohort, 8 out of 106 CpG sites across these genes showed nominal significant differential DNA methylation (Table S13), of which only cg11251498 (*IDO2*) was shown nominal significance for both MTG and AgeCoDe while the rest were not found in the MTG analysis. Further analysis, after adjusting for age and gender at both time points, revealed that the cg11251498 (*IDO2*) methylation level was significantly higher in AD converters compared to controls at baseline (*p* = 0.001). Although methylation levels were elevated in AD patients after a 4.5-year follow-up, this difference was not statistically significant (*p* = 0.051) (Table 2).

### *IDO2 *pyrosequencing in BBACL

In order to validate cg11251498 (*IDO2*), we pyrosequenced this locus in DNA from blood samples of subjects from an independent longitudinal cohort (BBACL). At baseline, after correcting for covariates, cg11251498 (*IDO2*) did not show a significant difference in DNA methylation levels when comparing SCD, MCI, and dementia patients (*model 1*, *p* = 0.58; *model 2*, *p* = 0.55). Similarly, no difference in *IDO2* DNA methylation was observed when comparing future converters and non-converters (MCI-D, MCI-MCI) at baseline (*model 1*, *p* = 0.38; *model 2*, *p* = 0.32) (Table 3). Interestingly, a strong negative association between *IDO2* DNA methylation and age was seen both when comparing SCD, MCI, and dementia patients (m*odel 1*: β = −0.11, S.E. = 0.04, *p* = 0.006, 95% CI = −0.19 to −0.031) and when comparing converters to non-converters (m*odel 1*: β = −0.14, S.E. = 0.053, *p* = 0.009, 95% CI = −0.24 to −0.035). Moreover, an age association was still significant for both comparisons when applying *model 2* (Table 5). Age significance was neither observed in AgeCoDe nor in the MTG data (Table S14). Finally, cg11251498 (*IDO2*) showed no significant association with cognition when comparing SCD, MCI, and dementia patients at baseline (*model 1*, *p* = 0.60; *model 2*, *p* = 0.15) and when comparing future converters and non-converters (*model 1*, *p* = 0.81; *model 2*, *p* = 0.30) (Table 5).

**Table 5:** *IDO2* methylation association with age and cognitive test in the BBACL cohort

		β	S.E.	t	*p*-value	95% CI	
**Baseline**	**Age**						
	*Model 1*^*a*^	−0.110	0.040	−2.750	0.006	−0.189	−0.031
	*Model 2*	−0.126	0.049	−2.558	0.011	−0.223	−0.029
	**normMMSE**						
	*Model 1*	−0.012	0.023	−0.519	0.604	−0.058	0.034
	*Model 2*	−0.042	0.029	−1.464	0.145	−0.099	0.015
**Converter**	**Age**						
	*Model 1*^*a*^	−0.139	0.053	−2.630	0.009	−0.244	−0.035
	*Model 2*	−0.163	0.067	−2.446	0.016	−0.296	−0.031
	**normMMSE**						
	*Model 1*	0.007	0.028	0.242	0.809	−0.048	0.061
	*Model 2*	−0.037	0.035	−1.042	0.300	−0.107	0.033

Association between *IDO2* percent methylation at position cg11251498 with age or normalized MMSE (normMMSE) for baseline diagnosis and in view of conversion within 76 months. *Model 1* adjusted for age, gender, education and diagnosis/converters, while *model 2* adjusted for *model 1* + lifestyle factors (tobacco use, alcohol use, and body mass index (BMI))

*Abbreviations:* *normMMSE* normalized mini mental state exam, *β* beta value, *S.E.* standard error, *t* t statistic, *95% CI* 95% confidence interval

^a^*Model 1* for age association adjusted for gender, education, and diagnosis/converters.

## Discussion

The present study aimed to investigate the TRP catabolic pathway and its potential dysregulation in the pathophysiology of AD. Making use of a selection of 60 TRP- and 57 NAD-associated genes, we conducted a hypothesis-driven pathway enrichment analysis, assessed gene-specific mRNA expression and DNA (hydroxy)methylation profiles, and performed a GRN and associated perturbation analysis, on brain tissue derived from AD patients and matched controls. We then validated the findings by zooming in on blood *IDO2* methylation in two independent longitudinal cohorts (AgeCoDe and BBACL).

For the TRP metabolic pathway, 12 genes showed significant differences in mRNA expression in the brains of patients with AD as compared to controls. TRP is an essential amino acid and transported from the periphery to the central nervous system (CNS) through an amino acid transporter. Based on our results, the transporter gene solute carrier family 7 member 5 (*SLC7A5)*, also known as the large amino acid transporter 1 (LAT1), showed a significant increase in mRNA expression, while exhibiting nominally significant differences at the level of 5hmC and 5uC, in AD. The KP is active under inflammatory conditions, where unbound free TRP competes with other large neutral amino acids (LNAAs) for the same LAT1 transporter. LAT1 is located in the capillaries of the blood-brain-barrier (BBB) and allows these amino acids to pass through the BBB, while LAT1 mRNA expression is (co)regulated by DNA methylation [36]. Taken together, these findings suggest that in AD, alterations in LAT1 expression led to an abnormal degree of delivery of TRP and other LNAAs to the CNS. Additionally, these expression differences by may be caused epigenetic regulation, although this latter aspect awaits further research.

Furthermore, *IDO2* displayed decreased mRNA expression and increased 5hmC and 5uC levels in AD. More importantly, both our GRN and network perturbation analysis pointed towards *IDO2* as a potentially critical player in the pathophysiology of AD. In fact, *IDO2* was part of the gene set with the highest perturbation score. In other words, *IDO2* is suggested to play a crucial role in the maintenance and stability of the phenotype under consideration and normalizing *IDO2* expression in AD has the potential to move the disease (AD) GRN towards that of healthy controls. While its exact function is still unclear, *IDO2* is structurally linked to and closely located near *IDO1* on chromosome 8. Studies have reported that IDO1 mediates T cell-suppressive effects, while IDO2 is a pro-inflammatory mediator of B cell responses and critical for IDO1-mediated T cell regulation [37, 38]. Studies have shown that under normal physiological or non-inflammatory conditions, IDO mRNA expression is either undetectable or very low, whereas upon immune stimulation, it is up-regulated significantly [39]. Neuroinflammation seems an important contributor to the pathophysiology of AD and is an early event in AD [40]. Interestingly, Guillemin et al. (2005) confirmed increased IDO activity in the AD hippocampus via immunohistochemistry [10]. The same group has previously shown that both inflammatory cytokines and Aβ can lead to increased cellular expression of IDO [41–43]. While no study was conducted on the therapeutic effects of IDO2 inhibitor, recent studies highlight the protective effects of IDO1 inhibitors such as DWG-1036 [44], PF0684003 [45], and RY103 [46] on AD pathology from preclinical models in both cell lines and mouse models. These inhibitors have been shown to improve behavioral and cognitive functions [44, 45], restore glucose metabolism within the hippocampus [45], and modulate the expression of key inflammatory cytokines and neurotrophic factors in this region [46]. Additionally, treatment with IDO1 inhibitor has led to upregulated expression of enzymes within the KP [46], suggesting a multifaceted therapeutic potential in addressing the complex neuroinflammatory and metabolic disruptions characteristic of AD.

Based on the MTG data, cg11251498 (*IDO2*) was selected as one of three potential candidate sites to be further validated in the blood of two independent longitudinal cohorts, i.e. AgeCoDe and BBACL. In AgeCoDe, at baseline, cg11251498 displayed a significant increase in methylation in those individuals subsequently converting to AD when compared to non-convertors. In BBACL, no significant difference in cg11251498 DNA methylation between SCD, MCI, and dementia patients was observed. However, a significant negative age association with cg11251498 methylation was seen in BBACL, an effect that in turn was neither observed in AgeCoDe nor in the MTG data. One plausible explanation for this apparent discrepancy as a function of age may be the different age ranges (MTG: age range 70–95 years, average age 85 years; AgeCoDe: 75–89 years, average age 82 years; BBACL: 43–90 years, average age 71 years) of individuals assessed in the various studies. Moreover, while the MTG and AgeCoDe studies both represent age-matched studies, for BBACL, SCD individuals were about 10 years younger than MCI and dementia patients. The exact link between *IDO2* methylation, age and AD, in the brain and blood, remains to be elucidated.

Tryptophan is also a precursor to the NAD pathway. In our transcriptomic analysis, 20 NAD pathway-associated genes showed significant differences in mRNA expression. Although the KP is often seen as the main driver for NAD production, recent studies suggest that the salvage pathway may be equally important in this respect [12, 47, 48]. Based on our MTG data, genes involved in the salvage pathways, especially poly (ADP-ribose) polymerases (PARPs), showed significant differences in gene expression. Studies have reported PARPs to be involved in DNA repair, cell proliferation, and cell death [49–51]. Amongst others, our data pointed towards *PARP14* both at the level of transcription and methylation, with cg14750551 displaying differential levels of 5mC and 5hmC, which correlated with *PARP14* mRNA expressions positively and negatively, respectively. Moreover, our GRN analysis showed that the *PARP14* node was linked to *PARP1* and *SIRT1*. Whereas no studies to date investigated the function of *PARP14* in AD, lipid studies have reported that *PAPR14* regulates low-density lipoproteins (LDL) receptors and apolipoproteins in macrophages, thus involved in hypercholesterolemia and hyperlipidemia [52]. Furthermore, studies have reported that cholesterol affects amyloid, tau, and gliosis in AD, and elevated LDL is a risk factor for developing AD [53, 54]. These results warrant further research into the role of these genes in AD.

The strength of this study was our stepwise approach starting off from post-mortem brain tissue to identify AD-associated differences in mRNA expression and DNA (hydroxy)methylation levels of TRP- and NAD-associated genes. The use of in silico modeling, including a GRN and network perturbation analysis allowed us to select the most suitable DNA methylation marks for validation in DNA derived from whole blood in two independent longitudinal aging cohorts. One challenge of the present study is the differences in design and group composition of the three cohorts. For example, the MTG dataset addresses Braak-associated changes in expression and methylation. For AgeCoDe, it starts off with healthy individuals of the same age, some of which over time develop AD, while BBACL included SCD, MCI, and dementia patients, all groups of which differed in age (which we have considered by adjusting for age in our main analyses). Of note, since we used bulk post-mortem MTG tissue, factors such as cell type composition, the vulnerability of specific cell types in AD and potential accompanying differences in cell-type proportions between groups, may have influenced the acquired data.

## Conclusion

Overall, our integrative analyses making use of post-mortem brain tissue and blood samples from two independent longitudinal cohorts have provided new information on the (dys)regulation and expression of genes involved in TRP- and NAD pathway-associated genes in AD. These findings provided first, preliminary evidence suggesting that *IDO2*, and more specifically cg11251498, could be a candidate biomarker for AD. While further research is required to elucidate the therapeutic potential of IDO2, the neuroprotective effects observed with IDO1 inhibition in AD pathology suggest that IDO2 may also serve as a promising therapeutic target. To conclude, transcriptional and epigenetic data analyses combined with in silico modeling represents a powerful tool to gain more insight into the involvement of key biological pathways in the development and course of AD.

## Supplementary Information

Supplementary Material 1.

## Data Availability

The datasets generated from the BSHRI-BBDP samples and analyzed during the current study are available in the Gene Expression Omnibus (GEO; https://www.ncbi.nlm.nih.gov/geo/) repository, under GEO accession numbers GSE109627 and GSE109887 for the epigenetic and expression data, respectively. The datasets generated from the AgeCoDe and BBACL samples and analyzed during the current study are not publicly available as participants did not provide informed consent for this, but are available from the corresponding author on reasonable request.

## Abbreviations

5hmC: 5-hydrxoymethycytosine
5mC: 5-methylcytosine
5uC: unmodified cytosine
AD: Alzheimer’s disease
AgeCoDe: The German Study of Ageing, Cognition, and Dementia in Primary Care Patients
BBACL: BioBank Alzheimer Center Limburg
GRN: Gene regulatory network
IDO: Indoleamine 2,3-dioxygenase
MTG: Middle temporal gyrus
NAD: Nicotinamide adenine dinucleotide
TRP: Tryptophan
