# Supplementary material for: Alzheimer’s disease-specific transcriptomic and epigenomic changes in the tryptophan catabolic pathway
Source: Alzheimers Res Ther. 2024 Nov 30;16:259. doi: 10.1186/s13195-024-01623-4 (PMC11607912; doi:10.1186/s13195-024-01623-4)
Supplement: Supplementary file 1 — Supplementary Material 1. [file 13195_2024_1623_MOESM1_ESM.docx]

**Alzheimer’s disease-specific transcriptomic and epigenomic changes in the tryptophan catabolic pathway**

Kyonghwan Choe^1^, Muhammad Ali^1,2,3^, Roy Lardenoije^1,4^, Renzo J.M. Riemens^1^, Ehsan Pishva^1,5^, Horst Bickel^6^, Siegfried Weyerer^7^, Per Hoffmann^8,9,10^, Michael Pentzek^11^, Steffi Riedel-Heller^12^, Birgitt Wiese^13^, Martin Scherer^14^, Michael Wagner^15,16^, Diego Mastroeni^1,17,18^, Paul D. Coleman^18^, Alfredo Ramirez^15,16,19,20,21^, Inez H.G.B. Ramakers^1,22^, Frans R.J. Verhey^1,22^, Bart P.F. Rutten^1^, Gunter Kenis^1^, and Daniel L.A. van den Hove^1*^

^1^Department of Psychiatry and Neuropsychology, Mental Health and Neuroscience Research Institute (MHeNs) and European Graduate School of Neuroscience (EURON), Faculty of Health, Medicine and Life Sciences (FHML), Maastricht University, Maastricht, the Netherlands.

^2^Computational Biology Group, Luxembourg Centre for System Biomedicine (LCSB), University of Luxembourg, Belvaux, Luxembourg.

^3^Biomedical Data science Group, Luxembourg Centre for System Biomedicine (LCSB), University of Luxembourg, Belvaux, Luxembourg.

^4^Pattern Recognition and Bioinformatics, Department of Intelligent Systems, Delft University of Technology, Delft, the Netherlands.

^5^University of Exeter Medical School, University of Exeter, Exeter, UK.

^6^Department of Psychiatry, Klinikum rechts der Isar, Technical University of Munich, Munich, Germany.

^7^Central Institute of Mental Health, Medical Faculty, Mannheim/Heidelberg University, Heidelberg, Germany.

^8^Institute of Human Genetics, University of Bonn, 53127 Bonn, Germany.

^9^Department of Genomics, Life & Brain Center, University of Bonn, 53127 Bonn, Germany.

^10^Division of Medical Genetics, University Hospital and Department of Biomedicine, University of Basel, CH-4058 Basel, Switzerland.

^11^Institute of General Practice, Medical Faculty, Heinrich Heine University Düsseldorf, 40225 Düsseldorf, Germany.

^12^Institute of Social Medicine, Occupational Health and Public Health, Leipzig University, 04103 Leipzig, Germany.

^13^Work Group Medical Statistics and IT-Infrastructure, Institute for General Practice, Hannover Medical School, Hannover, Germany.

^14^Department of Primary Medical Care, Center for Psychosocial Medicine, University Medical Center Hamburg-Eppendorf, Hamburg, Germany.

^15^Department of Neurodegeneration and Gerontopsychiatry, University of Bonn, 53127, Bonn, Germany.

^16^German Center for Neurodegenerative Diseases (DZNE), 53127 Bonn, Germany.

^17^L.J. Roberts Center for Alzheimer’s Research, Banner Sun Health Research Institute, Sun City, AZ, USA.

^18^Biodesign Institute, Neurodegenerative Disease Research Center, Arizona State University, Tempe, AZ, USA.

^19^Division of Neurogenetics and Molecular Psychiatry, Department of Psychiatry and Psychotherapy, University of Cologne, Medical Faculty, 50937 Cologne, Germany.

^20^Excellence Cluster on Cellular Stress Responses in Aging-Associated Diseases (CECAD) University of Cologne, Germany.

^21^Department of Psychiatry and Glenn Biggs Institute for Alzheimer’s and Neurodegenerative Diseases, San Antonio, TX, USA.

^22^Department of Psychiatry and Neuropsychology, Alzheimer Center Limburg, Maastricht University, Maastricht, the Netherlands.

***Corresponding author:**

Daniel L.A. van den Hove, PhD

Professor in Neuroepigenetics

Head Section Fundamental Neuroscience,

Department of Psychiatry and Neuropsychology,

Mental Health and Neuroscience Research Institute (MHeNs),

Maastricht University, 6200 MD Maastricht, the Netherlands

Tel.: +31-43-3882203

Email: [d.vandenhove@maastrichtuniversity.nl](mailto:d.vandenhove@maastrichtuniversity.nl)

**Supplemental Material**

**Table S1. List of TRP metabolic pathway associated genes**

|  | **Tryptophan metabolism pathway gene** | **Abbreviation** |
| --- | --- | --- |
| 1 | Aminoadipate Aminotransferase | AADAT |
| 2 | Aralkylamine N-Acetyltransferase | AANAT |
| 3 | Acetyl-CoA Acetyltransferase 1 | ACAT1 |
| 4 | Acetyl-CoA Acetyltransferase 2 | ACAT2 |
| 5 | Aminocarboxymuconate Semialdehyde Decarboxylase | ACMSD |
| 6 | Arylformamidase | AFMID |
| 7 | Aldehyde Dehydrogenase 1 Family Member A1 | ALDH1A1 |
| 8 | Aldehyde Dehydrogenase 1 Family Member A2 | ALDH1A2 |
| 9 | Aldehyde Dehydrogenase 1 Family Member B1 | ALDH1B1 |
| 10 | Aldehyde Dehydrogenase 2 Family Member | ALDH2 |
| 11 | Aldehyde Dehydrogenase 3 Family Member A2 | ALDH3A2 |
| 12 | Aldehyde Dehydrogenase 7 Family Member A1 | ALDH7A1 |
| 13 | Aldehyde Dehydrogenase 8 Family Member A1 | ALDH8A1 |
| 14 | Aldehyde Dehydrogenase 9 Family Member A1 | ALDH9A1 |
| 15 | Amine Oxidase, Copper Containing 1 | AOC1 |
| 16 | Aldehyde Oxidase 1 | AOX1 |
| 17 | Acetylserotonin O-Methyltransferase | ASMT |
| 18 | Catalase | CAT |
| 19 | Cytochrome P450 Family 19 Subfamily A Member 1 | CYP19A1 |
| 20 | Cytochrome P450 Family 1 Subfamily A Member 1 | CYP1A1 |
| 21 | Cytochrome P450 Family 1 Subfamily A Member 2 | CYP1A2 |
| 22 | Cytochrome P450 Family 1 Subfamily B Member 1 | CYP1B1 |
| 23 | Cytochrome P450 Family 2 Subfamily A Member 13 | CYP2A13 |
| 24 | Cytochrome P450 Family 2 Subfamily C Member 18 | CYP2C18 |
| 25 | Cytochrome P450 Family 2 Subfamily E Member 1 | CYP2E1 |
| 26 | Cytochrome P450 Family 2 Subfamily F Member 1 | CYP2F1 |
| 27 | Cytochrome P450 Family 2 Subfamily J Member 2 | CYP2J2 |
| 28 | Cytochrome P450 Family 3 Subfamily A Member 4 | CYP3A4 |
| 29 | Cytochrome P450 Family 4 Subfamily F Member 12 | CYP4F12 |
| 30 | Cytochrome P450 Family 7 Subfamily B Member 1 | CYP7B1 |
| 31 | Dopa Decarboxylase | DDC |
| 32 | 24-Dehydrocholesterol Reductase | DHCR24 |
| 33 | Dehydrogenase E1 And Transketolase Domain Containing 1 | DHTKD1 |
| 34 | Dihydrolipoamide Dehydrogenase | DLD |
| 35 | Dihydrolipoamide S-Succinyltransferase | DLST |
| 36 | Enoyl-CoA Hydratase, Short Chain 1 | ECHS1 |
| 37 | Enoyl-CoA Hydratase And 3-Hydroxyacyl CoA Dehydrogenase | EHHADH |
| 38 | Glutaryl-CoA Dehydrogenase | GCDH |
| 39 | Glutamic-Oxaloacetic Transaminase 2 | GOT2 |
| 40 | 3-Hydroxyanthranilate 3,4-Dioxygenase | HAAO |
| 41 | Hydroxyacyl-CoA Dehydrogenase | HADH |
| 42 | Hydroxyacyl-CoA Dehydrogenase Trifunctional Multienzyme Complex Subunit Alpha | HADHA |
| 43 | Indoleamine 2,3-Dioxygenase 1 | IDO1 |
| 44 | Indoleamine 2,3-Dioxygenase 2 | IDO2 |
| 45 | Interleukin 4 Induced 1 | IL4I1 |
| 46 | Indolethylamine N-Methyltransferase | INMT |
| 47 | Kynurenine 3-Monooxygenase | KMO |
| 48 | Kynurenine Aminotransferase 1 | KYAT1 |
| 49 | Kynurenine Aminotransferase 3 | KYAT3 |
| 50 | Kynureninase | KYNU |
| 51 | Monoamine Oxidase A | MAOA |
| 52 | Monoamine Oxidase B | MAOB |
| 53 | Oxoglutarate Dehydrogenase | OGDH |
| 54 | Solute Carrier Family 36 Member 4 | SLC36A4 |
| 55 | Solute Carrier Family 3 Member 2 | SLC3A2 |
| 56 | Solute Carrier Family 7 Member 5 | SLC7A5 |
| 57 | Tryptophan 2,3-Dioxygenase | TDO2 |
| 58 | Tryptophan Hydroxylase 1 | TPH1 |
| 59 | Tryptophan Hydroxylase 2 | TPH2 |
| 60 | Tryptophanyl-TRNA Synthetase | WARS |

**Table S2. List of NAD pathway associated genes**

|  | **Nicotinate and nicotinamide metabolism pathway gene** | **Abbreviation** |
| --- | --- | --- |
| 1 | Aminocarboxymuconate Semialdehyde Decarboxylase | ACMSD |
| 2 | Aldehyde Oxidase 1 | AOX1 |
| 3 | Bone Marrow Stromal Cell Antigen 1 | BST1 |
| 4 | CD38 Molecule | CD38 |
| 5 | Cytochrome P450 Family 8 Subfamily B Member 1 | CYP8B1 |
| 6 | Ectonucleotide Pyrophosphatase/Phosphodiesterase 1 | ENPP1 |
| 7 | Ectonucleotide Pyrophosphatase/Phosphodiesterase 3 | ENPP3 |
| 8 | Indoleamine 2,3-Dioxygenase 1 | IDO1 |
| 9 | NAD Kinase | NADK |
| 10 | NAD Kinase 2, Mitochondrial | NADK2 |
| 11 | NAD Synthetase 1 | NADSYN1 |
| 12 | Nicotinamide Phosphoribosyltransferase | NAMPT |
| 13 | Nicotinate Phosphoribosyltransferase | NAPRT |
| 14 | NAD(P)HX Dehydratase | NAXD |
| 15 | NAD(P)HX Epimerase | NAXE |
| 16 | Nicotinamide Nucleotide Adenylyltransferase 1 | NMNAT1 |
| 17 | Nicotinamide Nucleotide Adenylyltransferase 2 | NMNAT2 |
| 18 | Nicotinamide Nucleotide Adenylyltransferase 3 | NMNAT3 |
| 19 | Nicotinamide Riboside Kinase 1 | NMRK1 |
| 20 | Nicotinamide Riboside Kinase 2 | NMRK2 |
| 21 | Nicotinamide N-Methyltransferase | NNMT |
| 22 | Nicotinamide Nucleotide Transhydrogenase | NNT |
| 23 | Nik Related Kinase | NRK |
| 24 | 5', 3'-Nucleotidase, Cytosolic | NT5C |
| 25 | 5'-Nucleotidase, Cytosolic IA | NT5C1A |
| 26 | 5'-Nucleotidase, Cytosolic IB | NT5C1B |
| 27 | 5'-Nucleotidase, Cytosolic II | NT5C2 |
| 28 | 5'-Nucleotidase, Cytosolic IIIA | NT5C3A |
| 29 | 5'-Nucleotidase Ecto | NT5E |
| 30 | 5',3'-Nucleotidase, Mitochondrial | NT5M |
| 31 | Nudix Hydrolase 12 | NUDT12 |
| 32 | Poly(ADP-Ribose) Polymerase 1 | PAPR1 |
| 33 | Poly(ADP-Ribose) Polymerase 2 | PARP2 |
| 34 | Poly(ADP-Ribose) Polymerase 4 | PARP4 |
| 35 | Poly(ADP-Ribose) Polymerase 6 | PARP6 |
| 36 | Poly(ADP-Ribose) Polymerase 8 | PARP8 |
| 37 | Poly(ADP-Ribose) Polymerase 9 | PARP9 |
| 38 | Poly(ADP-Ribose) Polymerase 10 | PARP10 |
| 39 | Poly(ADP-Ribose) Polymerase 14 | PARP14 |
| 40 | Poly(ADP-Ribose) Polymerase 16 | PARP16 |
| 41 | Purine Nucleoside Phosphorylase | PNP |
| 42 | Prostaglandin I2 Synthase | PTGIS |
| 43 | Prostaglandin-Endoperoxide Synthase 2 | PTGS2 |
| 44 | Quinolinate Phosphoribosyltransferase | QPRT |
| 45 | Renalase, FAD Dependent Amine Oxidase | RNLS |
| 46 | Sirtuin 1 | SIRT1 |
| 47 | Sirtuin 2 | SIRT2 |
| 48 | Sirtuin 3 | SIRT3 |
| 49 | Sirtuin 4 | SIRT4 |
| 50 | Sirtuin 5 | SIRT5 |
| 51 | Sirtuin 6 | SIRT6 |
| 52 | Sirtuin 7 | SIRT7 |
| 53 | Solute Carrier Family 22 Member 13 | SLC22A13 |
| 54 | Solute Carrier Family 5 Member 8 | SLC5A8 |
| 55 | Tryptophan 2,3-Dioxygenase | TDO2 |
| 56 | Tankyrase | TNKS |
| 57 | Tankyrase 2 | TNKS2 |

**Table S3. *IDO2* PCR and sequencing primer overview**

| **Gene** | **Forward**  **Primer (5’-3’)** | **Reverse**  **primer (5’-3’)** | **Target region**  **(GRCh37)** | **Product size**  **(bp)** |
| --- | --- | --- | --- | --- |
| IDO2 | (Bio-)AGGAATTTTATA  ATAGAGAATAGTGATTT | ACCACCACAAAA  ATATTACATTTTCA | 8:39792704:39792853:1 | 150 |

| **Gene** | **Sequencing Primer** | **CpG** | **Target region**  **(GRCh37)** | **PyroMark**  **Orientation** |
| --- | --- | --- | --- | --- |
| IDO2 | CTAATAACTTCTTTTCTAACCT | 1 | 8:39792769: 39792774:1 | Lower stand  (5’-3’) |

The overview of the polymerase chain reaction (PCR) and pyrosequencing primers for *IDO2*. Abbreviation: Bio, biotinylation; GRCh37, Ensembl CRCh37 assembly; bp, base pair.

**Table S4. TRP metabolic pathway gene expression**

| **Gene Name** | **Fold**  **Change** | **AveExpr** | **t** | **S.E.** | **B** | **p-value** | **q-value**  **(FDR)** | **Associated pathway** |
| --- | --- | --- | --- | --- | --- | --- | --- | --- |
| TDO2 | 0.91 | 6.59 | -4.35 | 0.03 | 1.87 | <0.001 | 0.001 | Kynurenine |
| HAAO | 0.88 | 6.71 | -4.34 | 0.04 | 1.82 | <0.001 | 0.001 | Kynurenine |
| AOC1 | 0.91 | 6.63 | -4.18 | 0.03 | 1.30 | <0.001 | 0.001 | Tryptamine |
| GOT2 | 0.77 | 10.23 | -3.84 | 0.10 | 0.21 | <0.001 | 0.003 | Kynurenine |
| IDO2 | 0.94 | 6.61 | -3.83 | 0.02 | 0.15 | <0.001 | 0.003 | Kynurenine |
| DLD | 0.78 | 8.70 | -3.43 | 0.11 | -1.05 | 0.001 | 0.009 | Acetyl-CoA |
| SLC7A5 | 1.33 | 9.65 | 3.18 | 0.13 | -1.77 | 0.002 | 0.017 | Transporter |
| WARS | 0.84 | 8.75 | -3.11 | 0.08 | -1.95 | 0.003 | 0.018 | L-Tryptophanyl-tRNA |
| CYP2E1 | 0.85 | 7.30 | -3.08 | 0.08 | -2.02 | 0.003 | 0.018 | Serotonin/Melatonin |
| DHCR24 | 0.78 | 8.09 | -2.93 | 0.12 | -2.42 | 0.004 | 0.025 | 2-Oxoglutarate |
| DHTKD1 | 1.09 | 7.49 | 2.76 | 0.05 | -2.85 | 0.007 | 0.037 | Acetyl-CoA |
| KYAT3 | 0.90 | 7.48 | -2.68 | 0.06 | -3.05 | 0.009 | 0.042 | Kynurenine |
| ALDH8A1 | 1.10 | 7.09 | 2.59 | 0.05 | -3.26 | 0.012 | 0.05 | Acetyl-CoA |
| CYP2C18 | 0.98 | 6.49 | -2.49 | 0.01 | -3.49 | 0.015 | 0.06 | Serotonin/Melatonin |
| ECHS1 | 1.09 | 9.99 | 2.44 | 0.05 | -3.60 | 0.017 | 0.063 | Acetyl-CoA |
| CYP4F12 | 1.10 | 6.70 | 2.39 | 0.06 | -3.72 | 0.02 | 0.068 | Serotonin/Melatonin |
| AFMID | 0.93 | 7.04 | -2.35 | 0.04 | -3.81 | 0.022 | 0.071 | Kynurenine |
| ACMSD | 1.02 | 6.46 | 2.31 | 0.01 | -3.89 | 0.024 | 0.074 | Kynurenine |
| SLC3A2 | 1.13 | 8.46 | 2.19 | 0.08 | -4.14 | 0.032 | 0.091 | Transporter |
| ALDH1B1 | 0.96 | 6.86 | -2.16 | 0.03 | -4.20 | 0.034 | 0.091 | Serotonin |
| SLC36A4 | 1.13 | 8.25 | 2.14 | 0.08 | -4.25 | 0.036 | 0.091 | Transporter |
| ALDH1A1 | 0.83 | 9.26 | -2.12 | 0.13 | -4.27 | 0.037 | 0.091 | Serotonin/5-Methoxy-indoleacetate |
| ACAT2 | 0.87 | 7.82 | -2.12 | 0.10 | -4.28 | 0.037 | 0.091 | Acetyl-CoA |
| CYP19A1 | 1.03 | 6.62 | 2.07 | 0.02 | -4.37 | 0.042 | 0.096 | Serotonin/Melatonin |
| MAOB | 1.13 | 9.07 | 2.06 | 0.08 | -4.39 | 0.043 | 0.096 | Serotonin/4,6-Dihydroxy-quinoline |
| CAT | 1.17 | 8.50 | 2.03 | 0.11 | -4.45 | 0.046 | 0.097 | Kynurenine |
| CYP3A4 | 0.98 | 6.49 | -2.02 | 0.02 | -4.46 | 0.047 | 0.097 | Serotonin/Melatonin |
| CYP2F1 | 1.01 | 6.46 | 1.98 | 0.01 | -4.55 | 0.052 | 0.104 | Serotonin/Melatonin |
| CYP2J2 | 1.12 | 9.73 | 1.72 | 0.10 | -5.00 | 0.089 | 0.173 | Serotonin/Melatonin |
| IDO1 | 0.98 | 6.56 | -1.70 | 0.02 | -5.04 | 0.094 | 0.176 | Kynurenine |
| OGDH | 0.93 | 7.21 | -1.63 | 0.06 | -5.14 | 0.107 | 0.193 | Acetyl-CoA |
| ALDH2 | 1.11 | 10.90 | 1.56 | 0.10 | -5.25 | 0.123 | 0.216 | Serotonin/5-Methoxy-indoleacetate |
| INMT | 1.08 | 6.57 | 1.46 | 0.07 | -5.39 | 0.149 | 0.252 | Tryptamine & Serotonin/N-Methylserotonin |
| DLST | 1.06 | 7.61 | 1.43 | 0.06 | -5.44 | 0.158 | 0.253 | Acetyl-CoA |
| KYNU | 1.04 | 6.75 | 1.41 | 0.04 | -5.46 | 0.162 | 0.253 | Kynurenine |
| TPH2 | 0.99 | 6.58 | -1.41 | 0.02 | -5.46 | 0.163 | 0.253 | Serotonin |
| EHHADH | 0.96 | 7.43 | -1.27 | 0.05 | -5.65 | 0.21 | 0.318 | Acetyl-CoA |
| CYP1A1 | 1.02 | 6.59 | 1.21 | 0.02 | -5.71 | 0.23 | 0.339 | Serotonin/Melatonin |
| ALDH3A2 | 1.04 | 8.37 | 1.01 | 0.05 | -5.93 | 0.315 | 0.452 | Serotonin/5-Methoxy-indoleacetate & indoleacetate |
| KYAT1 | 1.05 | 8.34 | 0.92 | 0.07 | -6.01 | 0.359 | 0.502 | Kynurenine |
| ACAT1 | 0.95 | 9.45 | -0.85 | 0.08 | -6.07 | 0.396 | 0.537 | Acetyl-CoA |
| HADHA | 1.06 | 8.23 | 0.84 | 0.10 | -6.08 | 0.403 | 0.537 | Acetyl-CoA |
| MAOA | 1.06 | 10.59 | 0.78 | 0.11 | -6.13 | 0.436 | 0.559 | Serotonin/4,6-Dihydroxy-quinoline |
| ALDH9A1 | 1.03 | 11.59 | 0.78 | 0.06 | -6.13 | 0.44 | 0.559 | Serotonin/5-Methoxy-indoleacetate & indoleacetate |
| AADAT | 1.03 | 7.41 | 0.74 | 0.07 | -6.16 | 0.465 | 0.577 | Kynurenine |
| AOX1 | 1.03 | 6.56 | 0.72 | 0.05 | -6.17 | 0.474 | 0.577 | Serotonin/5-Methoxy-indoleacetate |
| ALDH1A2 | 0.97 | 6.81 | -0.70 | 0.06 | -6.19 | 0.485 | 0.577 | Serotonin/5-Methoxy-indoleacetate |
| CYP7B1 | 0.99 | 6.56 | -0.62 | 0.02 | -6.24 | 0.537 | 0.619 | 6-Hydroxyindolelactate |
| CYP1A2 | 1.00 | 6.44 | 0.61 | 0.01 | -6.24 | 0.542 | 0.619 | Serotonin/Melatonin |
| ALDH7A1 | 1.04 | 9.73 | 0.60 | 0.09 | -6.25 | 0.552 | 0.619 | Serotonin/5-Methoxy-indoleacetate & indoleacetate |
| DDC | 1.00 | 6.48 | -0.58 | 0.01 | -6.26 | 0.565 | 0.62 | Serotonin & Tryptamine |
| HADH | 0.98 | 9.92 | -0.39 | 0.07 | -6.35 | 0.696 | 0.75 | Acetyl-CoA |
| CYP2A13 | 1.00 | 6.54 | 0.35 | 0.01 | -6.37 | 0.726 | 0.767 | Serotonin/Melatonin |
| GCDH | 0.99 | 7.30 | -0.27 | 0.04 | -6.39 | 0.785 | 0.814 | Acetyl-CoA |
| IL4I1 | 1.00 | 6.48 | -0.17 | 0.02 | -6.42 | 0.865 | 0.881 | Indolepyruvate |
| ASMT | 1.00 | 6.45 | 0.11 | 0.01 | -6.42 | 0.914 | 0.914 | Serotonin/Melatonin/5-Methoxy-indoleacetate |
| AANAT | N/A | N/A | N/A | N/A | N/A | N/A | N/A | Kynurenine |
| CYP1B1 | N/A | N/A | N/A | N/A | N/A | N/A | N/A | Serotonin/Melatonin |
| KMO | N/A | N/A | N/A | N/A | N/A | N/A | N/A | Kynurenine |
| TPH1 | N/A | N/A | N/A | N/A | N/A | N/A | N/A | Serotonin |

Gene expression of TRP pathway associated genes in the middle temporal gyrus (MTG) in a comparison of Alzheimer’s disease (AD) patients and controls. Abbreviation: AveExpr, average expression; S.E., standard error; t, t-value; B, b value; FDR, false discovery rate.

**Table S5. NAD pathway gene expression**

| **Gene Name** | **Fold**  **Change** | **AveExpr** | **t** | **S.E.** | **B** | **p-**  **value** | **q-**  **value**  **(FDR)** | **Associated Pathway** |
| --- | --- | --- | --- | --- | --- | --- | --- | --- |
| SIRT1 | 1.21 | 8.63 | 5.91 | 0.05 | 7.63 | <0.001 | <0.001 | Salvage |
| NMNAT2 | 0.65 | 8.26 | -4.84 | 0.13 | 3.58 | <0.001 | <0.001 | Salvage & Preiss-Handler |
| PARP1 | 1.14 | 11.04 | 4.70 | 0.04 | 3.10 | <0.001 | <0.001 | Salvage |
| PARP9 | 1.25 | 7.65 | 4.37 | 0.07 | 1.94 | <0.001 | <0.001 | Salvage |
| TDO2 | 0.91 | 6.59 | -4.35 | 0.03 | 1.87 | <0.001 | <0.001 | De novo biosynthesis |
| PARP14 | 1.20 | 7.51 | 3.75 | 0.07 | -0.10 | <0.001 | 0.003 | Salvage |
| NT5C3A | 0.86 | 8.51 | -3.69 | 0.06 | -0.26 | <0.001 | 0.003 | Salvage & Preiss-Handler |
| PNP | 1.24 | 7.56 | 3.54 | 0.09 | -0.71 | 0.001 | 0.004 | Salvage & Preiss-Handler |
| SLC5A8 | 1.39 | 8.60 | 3.54 | 0.13 | -0.73 | 0.001 | 0.004 | Transporter |
| PARP4 | 1.26 | 8.39 | 3.49 | 0.10 | -0.86 | 0.001 | 0.004 | Salvage |
| QPRT | 1.21 | 8.32 | 3.34 | 0.08 | -1.31 | 0.001 | 0.006 | De novo biosynthesis |
| NT5C2 | 1.20 | 10.37 | 3.30 | 0.08 | -1.43 | 0.002 | 0.007 | Salvage & Preiss-Handler |
| PARP10 | 1.16 | 7.31 | 2.97 | 0.07 | -2.32 | 0.004 | 0.015 | Salvage |
| NAPRT | 1.27 | 8.37 | 2.94 | 0.12 | -2.40 | 0.004 | 0.015 | Preiss-Handler |
| NADSYN1 | 1.10 | 7.29 | 2.86 | 0.05 | -2.61 | 0.006 | 0.018 | Preiss-Handler |
| SIRT5 | 0.95 | 7.55 | -2.84 | 0.03 | -2.66 | 0.006 | 0.018 | Salvage |
| NT5M | 0.92 | 6.99 | -2.78 | 0.04 | -2.79 | 0.007 | 0.02 | Salvage & Preiss-Handler |
| NT5C | 1.16 | 8.53 | 2.68 | 0.08 | -3.06 | 0.009 | 0.026 | Salvage & Preiss-Handler |
| CYP8B1 | 0.98 | 6.50 | -2.54 | 0.01 | -3.37 | 0.013 | 0.035 | Salvage (endoplasmic reticulum lumen) |
| NADK | 1.08 | 7.31 | 2.49 | 0.04 | -3.49 | 0.015 | 0.038 | NADP+ |
| ACMSD | 1.02 | 6.46 | 2.31 | 0.01 | -3.89 | 0.024 | 0.057 | De novo biosynthesis |
| NNT | 0.85 | 8.71 | -2.29 | 0.11 | -3.94 | 0.025 | 0.058 | NADP+ |
| PTGIS | 0.97 | 6.55 | -2.23 | 0.02 | -4.05 | 0.029 | 0.063 | Salvage (endoplasmic reticulum lumen) |
| TNKS2 | 1.03 | 6.60 | 2.21 | 0.02 | -4.11 | 0.031 | 0.065 | Salvage |
| BST1 | 0.98 | 6.54 | -2.03 | 0.02 | -4.46 | 0.046 | 0.095 | Salvage |
| SIRT2 | 1.09 | 7.67 | 2.01 | 0.06 | -4.49 | 0.048 | 0.095 | Salvage |
| SIRT7 | 1.03 | 6.75 | 1.90 | 0.02 | -4.68 | 0.061 | 0.115 | Salvage |
| CD38 | 0.98 | 6.59 | -1.87 | 0.02 | -4.74 | 0.065 | 0.119 | Salvage |
| RNLS | 0.98 | 6.97 | -1.74 | 0.02 | -4.97 | 0.086 | 0.152 | Salvage (extracellular region) |
| IDO1 | 0.98 | 6.56 | -1.70 | 0.02 | -5.04 | 0.094 | 0.156 | De novo biosynthesis |
| NMNAT3 | 1.06 | 7.48 | 1.69 | 0.05 | -5.04 | 0.094 | 0.156 | Salvage & Preiss-Handler |
| NADK2 | 1.09 | 8.15 | 1.67 | 0.07 | -5.08 | 0.099 | 0.156 | NADP+ |
| NAMPT | 1.11 | 7.31 | 1.67 | 0.09 | -5.09 | 0.1 | 0.156 | Salvage |
| NT5C1B | 1.01 | 6.48 | 1.46 | 0.01 | -5.40 | 0.149 | 0.226 | Salvage & Preiss-Handler |
| ENPP1 | 1.03 | 6.76 | 1.40 | 0.03 | -5.48 | 0.167 | 0.246 | Salvage & Preiss-Handler |
| ENPP3 | 0.99 | 6.56 | -1.25 | 0.01 | -5.66 | 0.214 | 0.307 | Salvage & Preiss-Handler |
| PARP16 | 1.03 | 6.95 | 1.22 | 0.03 | -5.71 | 0.227 | 0.317 | Salvage |
| TNKS | 0.99 | 6.59 | -1.20 | 0.01 | -5.72 | 0.233 | 0.317 | Salvage |
| NMRK1 | 0.97 | 7.72 | -0.81 | 0.06 | -6.11 | 0.423 | 0.561 | Salvage & Preiss-Handler |
| SLC22A13 | 1.01 | 6.50 | 0.76 | 0.01 | -6.15 | 0.452 | 0.581 | Transporter |
| AOX1 | 1.03 | 6.56 | 0.72 | 0.05 | -6.17 | 0.474 | 0.581 | Salvage |
| NNMT | 0.99 | 6.58 | -0.72 | 0.02 | -6.18 | 0.476 | 0.581 | Salvage |
| SIRT6 | 0.99 | 6.63 | -0.71 | 0.03 | -6.18 | 0.483 | 0.581 | Salvage |
| NAXE | 0.98 | 8.96 | -0.63 | 0.06 | -6.23 | 0.531 | 0.626 | Salvage (mitochondrial matrix) |
| NT5C1A | 1.00 | 6.51 | -0.58 | 0.01 | -6.26 | 0.562 | 0.647 | Salvage & Preiss-Handler |
| PARP6 | 0.99 | 7.51 | -0.50 | 0.03 | -6.31 | 0.619 | 0.698 | Salvage |
| PTGS2 | 1.02 | 8.07 | 0.30 | 0.11 | -6.39 | 0.768 | 0.848 | Salvage (endoplasmic reticulum lumen) |
| NMRK2 | 1.00 | 6.48 | -0.24 | 0.01 | -6.40 | 0.808 | 0.857 | Salvage & Preiss-Handler |
| SIRT3 | 1.00 | 6.58 | 0.23 | 0.01 | -6.40 | 0.818 | 0.857 | Salvage |
| NAXD | 0.99 | 9.22 | -0.22 | 0.05 | -6.41 | 0.825 | 0.857 | Salvage (mitochondrial matrix) |
| NUDT12 | 1.00 | 6.62 | -0.13 | 0.03 | -6.42 | 0.894 | 0.912 | Salvage & Preiss-Handler |
| PARP8 | 1.00 | 6.48 | 0.07 | 0.01 | -6.43 | 0.946 | 0.946 | Salvage |
| NMNAT1 | N/A | N/A | N/A | N/A | N/A | N/A | N/A | Salvage & Preiss-Handler |
| NRK | N/A | N/A | N/A | N/A | N/A | N/A | N/A | Salvage |
| NT5E | N/A | N/A | N/A | N/A | N/A | N/A | N/A | Salvage & Preiss-Handler |
| PARP2 | N/A | N/A | N/A | N/A | N/A | N/A | N/A | Salvage |
| SIRT4 | N/A | N/A | N/A | N/A | N/A | N/A | N/A | Salvage |

Gene expression of NAD pathway associated genes in the middle temporal gyrus (MTG) in a comparison of Alzheimer’s disease (AD) patients and controls. Abbreviation: AveExpr, average expression; S.E., standard error; t, t-value; B, b value; FDR, false discovery rate.

**Table S6. Correlation between KP- and NAD-associated genes and AD pathologies**

|  | **Total Aβ plaque** | **Total tau tangle** |
| --- | --- | --- |
| **KP-associated genes** |  |  |
| TDO2 | -0.49 (-0.65 to -0.28)  **<0.0001** | -0.41 (-0.59 to -0.20)  **0.0003** |
| HAAO | -0.26 (-0.47 to -0.023)  0.0271 | -0.28 (-0.48 to -0.042)  0.0183 |
| GOT2 | -0.49 (-0.65 to -0.29)  **<0.0001** | -0.37 (-0.56 to -0.14)  **0.0014** |
| IDO2 | -0.31 (-0.51 to -0.079)  **0.0076** | -0.32 (-0.52 to -0.094)  **0.0052** |
| KYAT3 | -0.30 (-0.50 to -0.068)  **0.0099** | -0.24 (-0.45 to -0.0026)  0.0417 |
| **NAD-associated genes** |  |  |
| SIRT1 | 0.41 (0.19 to 0.59)  **0.0003** | 0.36 (0.13 to 0.55)  **0.0019** |
| NMNAT2 | -0.64 (-0.76 to -0.47)  **<0.0001** | -0.53 (-0.68 to -0.34)  **<0.0001** |
| PARP1 | 0.54 (0.35 to 0.69)  **<0.0001** | 0.34 (0.11 to 0.53)  0.0033 |
| PARP9 | 0.46 (0.25 to 0.63)  **<0.0001** | 0.36 (0.14 to 0.55)  **0.0017** |
| PARP14 | 0.47 (0.27 to 0.64)  **<0.0001** | 0.44 (0.22 to 0.61)  **0.0001** |
| NT5C3A | -0.50 (-0.66 to -0.30)  **<0.0001** | -0.44 (-0.61 to -0.23)  **<0.0001** |
| PNP | 0.37 (0.15 to 0.56)  **0.0011** | 0.32 (0.088 to 0.52)  0.0061 |
| SLC5A8 | 0.20 (-0.036 to 0.42)  0.0855 | 0.28 (0.044 to 0.48)  0.0174 |
| PARP4 | 0.37 (0.14 to 0.56)  **0.0014** | 0.31 (0.079 to 0.51)  0.0076 |
| QPRT | 0.43 (0.22 to 0.61)  **0.0001** | 0.38 (0.16 to 0.57)  **0.0009** |
| NT5C2 | 0.36 (0.14 to 0.55)  **0.0016** | 0.27 (0.040 to 0.48)  0.0188 |
| PARP10 | 0.47 (0.26 to 0.64)  **<0.0001** | 0.41 (0.20 to 0.59)  **0.0003** |
| NAPRT | 0.37 (0.15 to 0.56)  **0.0013** | 0.36 (0.13 to 0.55)  **0.0019** |
| NADSYN1 | 0.39 (0.17 to 0.57)  **0.0006** | 0.36 (0.13 to 0.55)  **0.0018** |
| SIRT5 | -0.25 (-0.46 to -0.012)  0.0348 | -0.26 (-0.47 to -0.024)  0.0267 |
| NT5M | -0.078 (-0.31 to 0.16)  0.5112 | -0.078 (-0.31 to 0.16)  0.5123 |
| NT5C | 0.31 (0.076 to 0.51)  0.0081 | 0.32 (0.094 to 0.52)  0.0053 |
| NADK | 0.41 (0.19 to 0.59)  **0.0003** | 0.35 (0.12 to 0.54)  **0.0027** |

Spearman correlation analysis between KP- and NAD-associated gene expressions and AD pathologies, total Aβ plaques (n = 73) and tau tangles (n = 73) in the middle temporal gyrus of patients with AD and age- and gender-matched controls. Data is presented as Spearman r (95% confidence interval) and p-value. Bold p-values indicate significance after Bonferroni correction (KP: p < 0.01; NAD: p < 0.0025).

**Table S7. TRP pathway 5mC levels**

| **Probe Name** | **Gene Name** | **Fold Change** | **AveExpr** | **t** | **S.E.** | **p-value** | **B** | **Chromosome** | **SNP position** | **Region** |
| --- | --- | --- | --- | --- | --- | --- | --- | --- | --- | --- |
| cg10216820 | TPH2 | 1.012 | 0.198 | 2.859 | 0.006 | 0.006 | -4.376 | 12 | 72332539 | TSS200 |
| cg09577907 | CYP19A1 | 1.021 | 0.704 | 2.704 | 0.011 | 0.008 | -4.771 | 15 | 51535668 | 5'UTR |
| cg08104579 | AANAT | 1.015 | 0.718 | 2.643 | 0.008 | 0.010 | -4.923 | 17 | 74467662 | 3'UTR;3'UTR |
| cg07887243 | MAOB | 0.990 | 0.066 | -2.587 | 0.006 | 0.012 | -5.058 | X | 43741530 | 1stExon |
| cg12978820 | CYP2A13 | 1.013 | 0.342 | 2.528 | 0.007 | 0.014 | -5.198 | 19 | 41593202 | TSS1500 |
| cg24406775 | SLC3A2 | 0.985 | 0.508 | -2.488 | 0.009 | 0.015 | -5.292 | 11 | 62655842 | Body |
| cg20352402 | ALDH8A1 | 1.026 | 0.531 | 2.434 | 0.015 | 0.017 | -5.417 | 6 | 135271333 | TSS200 |
| cg05903298 | ACAT1 | 0.999 | 0.020 | -2.419 | 0.001 | 0.018 | -5.450 | 11 | 107992155 | TSS200 |
| cg20336341 | DDC | 1.013 | 0.279 | 2.379 | 0.008 | 0.020 | -5.539 | 7 | 50628841 | TSS200;5'UTR |
| cg13198321 | ALDH1A2 | 0.998 | 0.019 | -2.275 | 0.001 | 0.026 | -5.768 | 15 | 58357891 | 5'UTR;1stExon;1stExon;5'UTR |
| cg18047172 | DDC | 1.014 | 0.552 | 2.240 | 0.009 | 0.028 | -5.844 | 7 | 50670059 | Body |
| cg13232821 | DDC | 1.015 | 0.334 | 2.221 | 0.010 | 0.029 | -5.883 | 7 | 50628718 | 5'UTR;5'UTR;1stExon |
| cg14080227 | ALDH1B1 | 0.997 | 0.052 | -2.176 | 0.002 | 0.033 | -5.979 | 9 | 38363157 |  |
| cg12715421 | DDC | 1.009 | 0.422 | 2.132 | 0.006 | 0.036 | -6.068 | 7 | 50629987 | 5'UTR;TSS1500 |
| cg00053769 | GOT2 | 1.011 | 0.845 | 2.103 | 0.007 | 0.039 | -6.128 | 16 | 58783589 |  |
| cg09207718 | CYP1A2 | 1.010 | 0.690 | 2.093 | 0.007 | 0.040 | -6.146 | 15 | 75041386 | 5'UTR |
| cg10649458 | MAOA | 1.014 | 0.819 | 2.072 | 0.009 | 0.042 | -6.188 | X | 43512216 |  |
| cg17952826 | ACAT2 | 1.025 | 0.278 | 2.024 | 0.017 | 0.047 | -6.283 | 6 | 160182184 | TSS1500 |
| cg18493449 | AANAT | 0.988 | 0.869 | -1.998 | 0.009 | 0.049 | -6.333 | 17 | 74467972 | Body |

List of nominal significant differentially methylated (5mC) probes in the TRP pathway associated gene in the middle temporal gyrus (MTG) in a comparison of Alzheimer’s disease (AD) patients and controls. Abbreviation: AveExpr, average expression; S.E., standard error; t, t-value; B, b value; TSS, transcription start site; 5’UTR, 5’untranslated region; 3’UTR, 3’untranslated region.

**Table S8. TRP pathway 5hmC levels**

| **Probe Name** | **Gene Name** | **Fold Change** | **AveExpr** | **t** | **S.E.** | **p-value** | **B** | **Chromosome** | **SNP position** | **Region** |
| --- | --- | --- | --- | --- | --- | --- | --- | --- | --- | --- |
| cg27214960 | CYP2E1 | 1.021 | 0.042 | 3.392 | 0.009 | 0.001 | -1.590 | 10 | 135343280 | Body |
| cg18125510 | WARS | 1.020 | 0.213 | 2.893 | 0.010 | 0.005 | -2.981 | 14 | 100841768 | 1stExon;TSS1500;5'UTR;  5'UTR;TSS1500;1stExon;  5'UTR;5'UTR |
| cg00565882 | CYP1B1 | 1.015 | 0.100 | 2.623 | 0.008 | 0.010 | -3.661 | 2 | 38300707 | Body |
| cg13134297 | INMT | 1.027 | 0.124 | 2.449 | 0.016 | 0.017 | -4.070 | 7 | 30737556 |  |
| cg09409405 | SLC7A5 | 0.986 | 0.045 | -2.310 | 0.009 | 0.024 | -4.379 | 16 | 87911198 |  |
| cg10268548 | ACAT2 | 1.012 | 0.159 | 2.243 | 0.007 | 0.028 | -4.524 | 6 | 160184041 | Body |
| cg13518442 | ALDH1A2 | 1.016 | 0.234 | 2.214 | 0.011 | 0.030 | -4.584 | 15 | 58358879 | TSS1500;TSS1500 |
| cg24406775 | SLC3A2 | 1.020 | 0.152 | 2.188 | 0.013 | 0.032 | -4.639 | 11 | 62655842 | Body |
| cg11363097 | IDO2 | 1.027 | 0.107 | 2.173 | 0.017 | 0.033 | -4.669 | 8 | 39792086 | TSS1500 |
| cg10169763 | SLC7A5 | 0.974 | 0.110 | -2.164 | 0.018 | 0.034 | -4.687 | 16 | 87873389 | Body |
| cg09577907 | CYP19A1 | 0.985 | 0.144 | -2.163 | 0.010 | 0.034 | -4.689 | 15 | 51535668 | 5'UTR;5'UTR |
| cg23532924 | DDC | 1.012 | 0.065 | 2.129 | 0.008 | 0.036 | -4.758 | 7 | 50633445 | TSS1500 |
| cg09767736 | IL4I1 | 1.015 | 0.156 | 2.059 | 0.010 | 0.043 | -4.898 | 19 | 50400119 | Body;1stExon;5'UTR |
| cg18285819 | INMT | 0.982 | 0.158 | -2.039 | 0.013 | 0.045 | -4.936 | 7 | 30736630 |  |
| cg01466330 | IDO2 | 1.012 | 0.056 | 2.039 | 0.008 | 0.045 | -4.936 | 8 | 39835697 | Body |
| cg00053769 | GOT2 | 0.989 | 0.0599 | -2.033 | 0.008 | 0.045 | -4.947 | 16 | 58783589 |  |
| cg27527503 | HADH | 1.021 | 0.231 | 2.033 | 0.015 | 0.045 | -4.948 | 4 | 108909664 | TSS1500 |
| cg04484695 | MAOB | 1.014 | 0.181 | 2.008 | 0.010 | 0.048 | -4.997 | X | 43742501 | TSS1500 |

List of nominal significant differentially hydroxymethylated (5hmC) probes in the TRP pathway associated gene in the middle temporal gyrus (MTG) in a comparison of Alzheimer’s disease (AD) patients and controls. Abbreviation: AveExpr, average expression; S.E., standard error; t, t-value; B, b value; TSS, transcription start site; 5’UTR, 5’untranslated region.

**Table S9. TRP pathway 5uC levels**

| **Probe Name** | **Gene Name** | **Fold Change** | **AveExpr** | **t** | **S.E.** | **p-value** | **B** | **Chromosome** | **SNP position** | **Region** |
| --- | --- | --- | --- | --- | --- | --- | --- | --- | --- | --- |
| cg00565882 | CYP1B1 | 0.981 | 0.778 | -3.067 | 0.009 | 0.003 | -3.750 | 2 | 38300707 | Body |
| cg18125510 | WARS | 0.978 | 0.682 | -3.034 | 0.010 | 0.003 | -3.838 | 14 | 100841768 | 1stExon;TSS1500;5'UTR;  5'UTR;TSS1500;1stExon;  5'UTR;5'UTR |
| cg10169763 | SLC7A5 | 1.025 | 0.242 | 2.992 | 0.012 | 0.004 | -3.953 | 16 | 87873389 | Body |
| cg20408276 | CYP1B1 | 0.971 | 0.328 | -2.888 | 0.015 | 0.005 | -4.230 | 2 | 38300586 | Body |
| cg13472594 | ALDH1B1 | 0.986 | 0.474 | -2.871 | 0.007 | 0.005 | -4.275 | 9 | 38346777 |  |
| cg04790887 | ALDH1A2 | 0.987 | 0.160 | -2.626 | 0.007 | 0.010 | -4.894 | 15 | 58515544 |  |
| cg19571004 | CYP2E1 | 0.982 | 0.250 | -2.566 | 0.010 | 0.012 | -5.038 | 10 | 135340850 | TSS200 |
| cg13857519 | TPH1 | 0.994 | 0.088 | -2.556 | 0.004 | 0.013 | -5.062 | 11 | 18062735 | TSS1500 |
| cg20352402 | ALDH8A1 | 0.981 | 0.213 | -2.505 | 0.011 | 0.014 | -5.182 | 6 | 135271333 | TSS200 |
| cg14225090 | ALDH1B1 | 0.993 | 0.091 | -2.441 | 0.004 | 0.017 | -5.330 | 9 | 38237796 |  |
| cg04582695 | AANAT | 0.984 | 0.282 | -2.435 | 0.009 | 0.017 | -5.344 | 17 | 74449757 | 5'UTR;1stExon;TSS1500 |
| cg10649458 | MAOA | 0.989 | 0.124 | -2.360 | 0.007 | 0.021 | -5.514 | X | 43512216 |  |
| cg08645207 | AOX1 | 0.976 | 0.296 | -2.285 | 0.015 | 0.025 | -5.678 | 2 | 201489698 | Body |
| cg03236170 | SLC7A5 | 0.976 | 0.570 | -2.247 | 0.016 | 0.028 | -5.758 | 16 | 87915396 |  |
| cg16738971 | ALDH7A1 | 0.994 | 0.923 | -2.238 | 0.004 | 0.028 | -5.779 | 5 | 125931166 | TSS200 |
| cg01052699 | AADAT | 0.991 | 0.668 | -2.235 | 0.006 | 0.028 | -5.785 | 4 | 171030995 |  |
| cg02162897 | CYP1B1 | 0.984 | 0.211 | -2.233 | 0.010 | 0.029 | -5.789 | 2 | 38300537 | Body |
| cg06264984 | CYP1B1 | 0.988 | 0.763 | -2.208 | 0.008 | 0.030 | -5.841 | 2 | 38300885 | Body |
| cg22874188 | AOC1 | 0.992 | 0.125 | -2.141 | 0.005 | 0.036 | -5.980 | 7 | 150555302 | Body |
| cg16437184 | ALDH8A1 | 0.982 | 0.314 | -2.136 | 0.012 | 0.036 | -5.990 | 6 | 135157459 |  |
| cg12802310 | CYP1B1 | 0.988 | 0.594 | -2.122 | 0.008 | 0.037 | -6.019 | 2 | 38304720 | TSS1500 |
| cg07887243 | MAOB | 1.009 | 0.929 | 2.119 | 0.006 | 0.037 | -6.026 | X | 43741530 | 1stExon |
| cg11251498 | IDO2 | 0.981 | 0.304 | -2.077 | 0.014 | 0.041 | -6.109 | 8 | 39792769 | Body |
| cg20336341 | DDC | 0.989 | 0.641 | -2.065 | 0.008 | 0.042 | -6.133 | 7 | 50628841 | TSS200;5'UTR |
| cg18872418 | TPH1 | 0.989 | 0.149 | -2.062 | 0.008 | 0.043 | -6.140 | 11 | 18068355 |  |
| cg13198321 | ALDH1A2 | 1.002 | 0.977 | 2.037 | 0.001 | 0.045 | -6.188 | 15 | 58357891 | 5'UTR;1stExon;1stExon;5'UTR |
| cg01812894 | ALDH1A1 | 0.987 | 0.189 | -2.017 | 0.009 | 0.047 | -6.227 | 9 | 75568506 | TSS1500 |
| cg25924411 | GCDH | 0.998 | 0.955 | -2.009 | 0.002 | 0.048 | -6.243 | 19 | 13001836 | TSS200 |
| cg10335743 | DDC | 0.995 | 0.091 | -2.002 | 0.004 | 0.049 | -6.256 | 7 | 50634611 | TSS1500 |

List of nominal significant differentially unmodified (5uC) probes in the TRP pathway associated gene in the middle temporal gyrus (MTG) in a comparison of Alzheimer’s disease (AD) patients and controls. Abbreviation: AveExpr, average expression; S.E., standard error; t, t-value; B, b value; TSS, transcription start site; 5’UTR, 5’untranslated region.

**Table S10. NAD pathway 5mC levels**

| **Probe Name** | **Gene Name** | **Fold Change** | **AveExpr** | **t** | **S.E.** | **p-value** | **B** | **Chromosome** | **SNP position** | **Region** |
| --- | --- | --- | --- | --- | --- | --- | --- | --- | --- | --- |
| cg17766305 | RNLS | 0.989 | 0.111 | -2.896 | 0.006 | 0.005 | -4.280 | 10 | 90147030 | Body |
| cg07612655 | PTGIS | 0.986 | 0.288 | -2.821 | 0.007 | 0.006 | -4.476 | 20 | 48185517 | TSS1500 |
| cg26707013 | SIRT4 | 1.030 | 0.516 | 2.635 | 0.016 | 0.010 | -4.943 | 12 | 120752270 |  |
| cg21580588 | NADK | 1.006 | 0.967 | 2.492 | 0.003 | 0.015 | -5.281 | 1 | 1685723 | Body |
| cg07595035 | NAXD | 1.025 | 0.595 | 2.389 | 0.015 | 0.019 | -5.518 | 13 | 111291582 | 3'UTR |
| cg21464278 | PARP9 | 0.997 | 0.045 | -2.358 | 0.002 | 0.021 | -5.586 | 3 | 122283593 | TSS1500;TSS1500;TSS200;  TSS1500;Body;TSS200;  TSS1500 |
| cg08378505 | NT5C | 0.999 | 0.022 | -2.319 | 0.001 | 0.023 | -5.673 | 17 | 73127297 | Body |
| cg09185911 | NADK | 1.018 | 0.808 | 2.284 | 0.011 | 0.025 | -5.749 | 1 | 1688883 | Body |
| cg15260978 | NADK | 1.022 | 0.612 | 2.281 | 0.014 | 0.025 | -5.755 | 1 | 1685832 | Body |
| cg12135976 | SLC5A8 | 1.010 | 0.116 | 2.255 | 0.006 | 0.027 | -5.811 | 12 | 101604147 | TSS200 |
| cg04444303 | SIRT1 | 0.978 | 0.779 | -2.252 | 0.014 | 0.027 | -5.818 | 10 | 69634106 |  |
| cg10582690 | SIRT1 | 0.999 | 0.021 | -2.229 | 0.001 | 0.029 | -5.866 | 10 | 69644422 | TSS1500;TSS200 |
| cg12488274 | PARP16 | 0.975 | 0.580 | -2.189 | 0.017 | 0.032 | -5.950 | 15 | 65594642 |  |
| cg20613259 | NT5C2 | 1.019 | 0.411 | 2.149 | 0.012 | 0.035 | -6.034 | 10 | 104868306 | Body |
| cg25038311 | NT5C2 | 1.018 | 0.499 | 2.125 | 0.012 | 0.037 | -6.082 | 10 | 104964751 |  |
| cg11229284 | PARP14 | 0.996 | 0.065 | -2.113 | 0.003 | 0.038 | -6.106 | 3 | 122399506 | TSS200 |
| cg08122652 | PARP9 | 1.015 | 0.472 | 2.081 | 0.010 | 0.041 | -6.170 | 3 | 122281939 | 5'UTR;5'UTR;TSS1500;  5'UTR;5'UTR;5'UTR;  5'UTR |
| cg09321403 | SIRT5 | 1.011 | 0.112 | 2.077 | 0.008 | 0.041 | -6.179 | 6 | 13555624 |  |
| cg14750551 | PARP14 | 1.023 | 0.534 | 2.029 | 0.016 | 0.046 | -6.273 | 3 | 122401343 | Body |
| cg20754155 | NAXD | 1.011 | 0.809 | 2.013 | 0.008 | 0.048 | -6.304 | 13 | 111256586 |  |

List of nominal significant differentially methylated (5mC) probes in the NAD pathway associated gene in the middle temporal gyrus (MTG) in a comparison of Alzheimer’s disease (AD) patients and controls. Abbreviation: AveExpr, average expression; S.E., standard error; t, t-value; B, b value; TSS, transcription start site; 5’UTR, 5’untranslated region; 3’UTR, 3’untranslated region.

**Table S11. NAD pathway 5hmC levels**

| **Probe Name** | **Gene Name** | **Fold Change** | **AveExpr** | **t** | **S.E.** | **p-value** | **B** | **Chromosome** | **SNP position** | **Region** |
| --- | --- | --- | --- | --- | --- | --- | --- | --- | --- | --- |
| cg07612655 | PTGIS | 1.026 | 0.218 | 4.164 | 0.009 | 0.000 | 0.874 | 20 | 48185517 | TSS1500 |
| cg24937136 | PARP1 | 1.022 | 0.382 | 3.137 | 0.010 | 0.002 | -2.323 | 1 | 226593346 | Body |
| cg03530744 | NAXD | 1.025 | 0.332 | 2.965 | 0.012 | 0.004 | -2.793 | 13 | 111270991 | Body |
| cg20819482 | TNKS | 1.035 | 0.303 | 2.867 | 0.017 | 0.005 | -3.049 | 8 | 9472057 | Body |
| cg23809813 | RNLS | 1.018 | 0.352 | 2.793 | 0.009 | 0.007 | -3.240 | 10 | 90217672 | Body |
| cg25147026 | PTGS2 | 1.024 | 0.273 | 2.725 | 0.013 | 0.008 | -3.412 | 1 | 186650441 | TSS1500 |
| cg01683055 | PARP4 | 1.018 | 0.041 | 2.689 | 0.010 | 0.009 | -3.501 | 13 | 25026835 | Body |
| cg26707013 | SIRT4 | 0.974 | 0.347 | -2.365 | 0.016 | 0.021 | -4.259 | 12 | 120752270 |  |
| cg13415078 | NAXD | 0.984 | 0.045 | -2.363 | 0.010 | 0.021 | -4.264 | 13 | 111287117 | Body |
| cg21580588 | NADK | 0.994 | 0.017 | -2.349 | 0.004 | 0.021 | -4.295 | 1 | 1685723 | Body |
| cg05722993 | NADK | 1.018 | 0.331 | 2.329 | 0.011 | 0.023 | -4.339 | 1 | 1727796 | Body |
| cg14750551 | PARP14 | 0.975 | 0.335 | -2.294 | 0.016 | 0.025 | -4.414 | 3 | 122401343 | Body |
| cg27058217 | NAXD | 1.017 | 0.121 | 2.252 | 0.011 | 0.027 | -4.504 | 13 | 111289041 | Body |
| cg03081930 | NAXD | 0.981 | 0.173 | -2.214 | 0.013 | 0.030 | -4.584 | 13 | 111288583 | Body |
| cg09560636 | NNT | 1.022 | 0.156 | 2.096 | 0.015 | 0.039 | -4.824 | 5 | 43631579 | Body |
| cg15897209 | NNMT | 1.018 | 0.267 | 2.074 | 0.013 | 0.041 | -4.867 | 11 | 114151397 |  |
| cg15824543 | NADK | 1.021 | 0.199 | 2.074 | 0.015 | 0.041 | -4.868 | 1 | 1695391 | Body |
| cg24867653 | SLC22A13 | 0.993 | 0.054 | -2.024 | 0.005 | 0.046 | -4.966 | 3 | 38306594 | TSS1500 |

List of nominal significant differentially hydroxymethylated (5hmC) probes in the NAD pathway associated gene in the middle temporal gyrus (MTG) in a comparison of Alzheimer’s disease (AD) patients and controls. Abbreviation: AveExpr, average expression; S.E., standard error; t, t-value; B, b value; TSS, transcription start site.

**Table S12. NAD pathway 5uC levels**

| **Probe Name** | **Gene Name** | **Fold Change** | **AveExpr** | **t** | **S.E.** | **p-value** | **B** | **Chromosome** | **SNP position** | **Region** |
| --- | --- | --- | --- | --- | --- | --- | --- | --- | --- | --- |
| cg03530744 | NAXD | 0.982 | 0.184 | -3.410 | 0.008 | 0.001 | -2.762 | 13 | 111270991 | Body |
| cg06516476 | NT5E | 1.016 | 0.343 | 3.406 | 0.007 | 0.001 | -2.775 | 6 | 86174584 | Body |
| cg16540590 | NNT | 0.980 | 0.193 | -2.963 | 0.010 | 0.004 | -4.032 | 5 | 43802496 |  |
| cg05238288 | PARP4 | 0.992 | 0.933 | -2.869 | 0.004 | 0.005 | -4.279 | 13 | 25087299 | TSS1500 |
| cg24647015 | NNMT | 0.991 | 0.131 | -2.645 | 0.005 | 0.010 | -4.848 | 11 | 114191367 |  |
| cg07612655 | PTGIS | 0.988 | 0.493 | -2.634 | 0.007 | 0.010 | -4.875 | 20 | 48185517 | TSS1500 |
| cg25038311 | NT5C2 | 0.979 | 0.340 | -2.532 | 0.012 | 0.013 | -5.120 | 10 | 104964751 |  |
| cg19966621 | NT5C3A | 1.003 | 0.970 | 2.513 | 0.002 | 0.014 | -5.163 | 7 | 33102440 | TSS200 |
| cg05722993 | NADK | 0.985 | 0.230 | -2.468 | 0.009 | 0.016 | -5.267 | 1 | 1727796 | Body |
| cg17130251 | NMRK2 | 0.985 | 0.342 | -2.459 | 0.009 | 0.016 | -5.289 | 19 | 3945936 |  |
| cg20819482 | TNKS | 0.969 | 0.382 | -2.408 | 0.019 | 0.019 | -5.406 | 8 | 9472057 | Body |
| cg09546332 | PARP4 | 0.997 | 0.941 | -2.388 | 0.002 | 0.019 | -5.451 | 13 | 25086777 | 5'UTR |
| cg02977388 | RNLS | 0.974 | 0.599 | -2.319 | 0.017 | 0.023 | -5.605 | 10 | 90253615 | Body |
| cg07631435 | NNMT | 0.984 | 0.723 | -2.315 | 0.010 | 0.023 | -5.613 | 11 | 114043903 | Body |
| cg26176158 | NT5C2 | 1.004 | 0.052 | 2.289 | 0.003 | 0.025 | -5.670 | 10 | 104936358 | 5'UTR |
| cg08645207 | AOX1 | 0.976 | 0.296 | -2.285 | 0.015 | 0.025 | -5.678 | 2 | 201489698 | Body |
| cg13215387 | NT5C2 | 0.998 | 0.966 | -2.273 | 0.001 | 0.026 | -5.704 | 10 | 104953220 | TSS200 |
| cg16373880 | NMNAT2 | 0.990 | 0.160 | -2.270 | 0.006 | 0.026 | -5.710 | 1 | 183274638 | Body;TSS1500 |
| cg11592576 | CYP8B1 | 0.984 | 0.273 | -2.261 | 0.010 | 0.027 | -5.730 | 3 | 42915467 | 3'UTR;1stExon |
| cg05262549 | NMNAT2 | 0.989 | 0.775 | -2.206 | 0.008 | 0.030 | -5.846 | 1 | 183256588 | Body |
| cg05215649 | NADSYN1 | 0.989 | 0.306 | -2.191 | 0.007 | 0.032 | -5.878 | 11 | 71196591 | Body |
| cg19518845 | NMNAT2 | 1.015 | 0.206 | 2.190 | 0.010 | 0.032 | -5.880 | 1 | 183338346 | Body |
| cg09936839 | SIRT6 | 0.985 | 0.466 | -2.168 | 0.010 | 0.033 | -5.925 | 19 | 4181854 | Body;TSS1500 |
| cg14098385 | PARP9 | 0.994 | 0.107 | -2.166 | 0.004 | 0.034 | -5.929 | 3 | 122285010 | TSS1500;TSS1500;Body |
| cg08122070 | NADK | 1.007 | 0.096 | 2.153 | 0.005 | 0.035 | -5.956 | 1 | 1689610 | Body |
| cg26802917 | NRK | 0.991 | 0.126 | -2.148 | 0.006 | 0.035 | -5.965 | X | 105064008 |  |
| cg01749742 | NT5C1B | 0.981 | 0.462 | -2.131 | 0.013 | 0.036 | -6.000 | 2 | 18944047 |  |
| cg08482694 | PTGS2 | 0.998 | 0.960 | -2.106 | 0.002 | 0.039 | -6.052 | 1 | 186649530 | 5'UTR;1stExon |
| cg03315247 | PARP14 | 0.996 | 0.937 | -2.093 | 0.003 | 0.040 | -6.078 | 3 | 122399498 | TSS200 |
| cg22566518 | SIRT6 | 0.997 | 0.951 | -2.074 | 0.002 | 0.042 | -6.115 | 19 | 4182796 | TSS1500 |
| cg22930808 | PARP9 | 0.989 | 0.173 | -2.059 | 0.008 | 0.043 | -6.145 | 3 | 122281881 | 5'UTR;5'UTR;TSS1500;  5'UTR;5'UTR;5'UTR;  5'UTR |
| cg21356631 | BST1 | 0.978 | 0.380 | -2.043 | 0.015 | 0.045 | -6.177 | 4 | 15702461 |  |
| cg06371014 | NMRK2 | 0.992 | 0.176 | -2.034 | 0.006 | 0.046 | -6.194 | 19 | 3924433 | Body |
| cg08200577 | PARP4 | 0.992 | 0.891 | -2.017 | 0.006 | 0.047 | -6.228 | 13 | 25086393 | 5'UTR |
| cg12488274 | PARP16 | 1.012 | 0.324 | 2.009 | 0.009 | 0.048 | -6.242 | 15 | 65594642 |  |

List of nominal significant differentially unmodified (5uC) probes in the NAD pathway associated gene in the middle temporal gyrus (MTG) in a comparison of Alzheimer’s disease (AD) patients and controls. Abbreviation: AveExpr, average expression; S.E., standard error; t, t-value; B, b value; TSS, transcription start site; 5’UTR, 5’untranslated region; 3’UTR, 3’untranslated region.

**Table S13. AgeCoDe baseline 5mC levels**

| **Probe Name** | **Gene Name** | **Fold Change** | **AveExpr** | **t** | **S.E.** | **p-value** | **Region** |
| --- | --- | --- | --- | --- | --- | --- | --- |
| cg06492111 | SLC7A5 | 0.995 | 0.962 | -3.412 | 0.002 | 0.001 | Body |
| cg26637881 | SLC7A5 | 0.995 | 0.901 | -2.539 | 0.003 | 0.011 | Body |
| cg22972380 | PARP14 | 1.004 | 0.111 | 2.496 | 0.002 | 0.013 | TSS200 |
| cg06770731 | SLC7A5 | 0.995 | 0.950 | -2.335 | 0.003 | 0.020 | 3'UTR |
| cg05393733 | SLC7A5 | 0.996 | 0.914 | -2.310 | 0.003 | 0.021 | Body |
| cg11251498 | IDO2 | 1.015 | 0.742 | 2.268 | 0.010 | 0.023 | Body |
| cg10520099 | SLC7A5 | 1.006 | 0.888 | 2.226 | 0.004 | 0.026 |  |
| cg09391488 | SLC7A5 | 1.015 | 0.163 | 2.015 | 0.011 | 0.044 |  |

List of nominal significant differentially methylated (5mC) probes, based on the candidate genes from the MTG dataset, in blood samples comparing Alzheimer’s disease (AD) converters and non-converters at baseline. Abbreviation: MTG, middle temporal gyrus; AveExpr, average expression; S.E., standard error; t, t-value; TSS, transcription start site; 3’UTR, 3’untranslated region.

**Table S14. AgeCoDe and MTG *IDO2* methylation association with age**

|  | **β** | **S.E.** | **t** | **p-value** | **95% CI** | |
| --- | --- | --- | --- | --- | --- | --- |
| **AgeCoDe** |  |  |  |  |  | |
| Baseline | -0.149 | 0.139 | -1.075 | 0.285 | -0.424 | 0.126 |
| Follow-ups | -0.137 | 0.166 | -0.825 | 0.412 | -0.467 | 0.193 |
| **MTG** |  |  |  |  |  |  |
| Transcriptomic | -0.001 | 0.002 | -0.285 | 0.777 | -0.004 | 0.003 |
| 5mC | 0.001 | 0.001 | 0.892 | 0.375 | -0.001 | 0.002 |
| 5hmC | 7.097E-5 | 0.001 | 0.064 | 0.949 | -0.002 | 0.002 |
| 5uC | -0.001 | 0.001 | -1.026 | 0.308 | -0.003 | 0.001 |

Association between *IDO2* percent methylation at position cg11251498 with age in AgeCoDe baseline and follow-up, and the middle temporal gyrus (MTG). Both analyses adjusted gender and diagnosis stage. Abbreviations: MTG, middle temporal gyrus; β, beta value; S.E., standard error; t, t statistic; 95% CI, 95% confidence interval.
